# The type of stress matters: repeated injection and permanent social isolation stress in male mice have a differential effect on anxiety- and depressive-like behaviours, and associated biological alterations

**DOI:** 10.1038/s41398-020-01000-3

**Published:** 2020-09-21

**Authors:** Andrea Du Preez, Thomas Law, Diletta Onorato, Yau M. Lim, Paola Eiben, Ksenia Musaelyan, Martin Egeland, Abdul Hye, Patricia A. Zunszain, Sandrine Thuret, Carmine M. Pariante, Cathy Fernandes

**Affiliations:** 1 Department of Basic and Clinical Neuroscience, Institute of Psychiatry, Psychology & Neuroscience, King’s College London, London, UK; 2 Department of Psychological Medicine, Institute of Psychiatry, Psychology & Neuroscience, King’s College London, London, UK; 3 Department of Old Age Psychiatry, Institute of Psychiatry, Psychology & Neuroscience, King’s College London, London, UK; 4 Social, Genetic & Developmental Psychiatry Centre, Institute of Psychiatry, Psychology & Neuroscience, King’s College London, London, UK; 5 MRC Centre for Neurodevelopmental Disorders, Institute of Psychiatry, Psychology & Neuroscience, King’s College London, London, UK

**Keywords:** Pathogenesis, Neuroscience

## Abstract

Chronic stress can alter the immune system, adult hippocampal neurogenesis and induce anxiety- and depressive-like behaviour in rodents. However, previous studies have not discriminated between the effect(s) of different types of stress on these behavioural and biological outcomes. We investigated the effect(s) of repeated injection vs. permanent social isolation on behaviour, stress responsivity, immune system functioning and hippocampal neurogenesis, in young adult male mice, and found that the type of stress exposure does indeed matter. Exposure to 6 weeks of repeated injection resulted in an anxiety-like phenotype, decreased systemic inflammation (i.e., reduced plasma levels of TNFα and IL4), increased corticosterone reactivity, increased microglial activation and decreased neuronal differentiation in the dentate gyrus (DG). In contrast, exposure to 6 weeks of permanent social isolation resulted in a depressive-like phenotype, increased plasma levels of TNFα, decreased plasma levels of IL10 and VEGF, decreased corticosterone reactivity, decreased microglial cell density and increased cell density for radial glia, s100β-positive cells and mature neuroblasts—all in the DG. Interestingly, combining the two distinct stress paradigms did not have an additive effect on behavioural and biological outcomes, but resulted in yet a different phenotype, characterized by increased anxiety-like behaviour, decreased plasma levels of IL1β, IL4 and VEGF, and decreased hippocampal neuronal differentiation, without altered neuroinflammation or corticosterone reactivity. These findings demonstrate that different forms of chronic stress can differentially alter both behavioural and biological outcomes in young adult male mice, and that combining multiple stressors may not necessarily cause more severe pathological outcomes.

## Introduction

Animal research has been paramount in investigating the association between stress and major depressive disorder (MDD)^1^. Exposure to individual or multiple stressors in rodents can alter the immune system^2,3^ and the hypothalamic–pituitary–adrenal (HPA) axis^4,5^, decrease hippocampal neurogenesis^6,7^ and induce anxiety- and depressive-like behaviours^8,9^. These areas of research have predominately utilized either (i) unpredictable chronic mild stress (UCMS) models, which incorporate a variety of both physical and psychosocial stressors^10^, or (ii) a social-stress-based model (e.g., social isolation or social defeat stress), which employ predominately psychosocial stressors^11,12^. However, one main limitation to this area of research is that there is no discrimination between the types of stress used, as most studies predominately incorporate either one or a combination of both these stress types. Thus, the distinction between the effect of different types of stress on animal behaviour and physiology has been largely unexplored. This idea is not entirely novel in a clinical setting, with research showing that psychological, sexual and physical maltreatment can differentially affect mental health^13–16^. For example, Hodgdon et al.^15^ recently demonstrated that psychological, sexual and physical abuse lead to distinct behavioural profiles and this research warrants being investigated in a preclinical setting, which, to our knowledge, is yet to be done.

Thus, is one type of stress exposure more potent than another and, if so, does this matter? This has already been partially addressed by a few preclinical studies showing how only UCMS and/or predator stress, but not restraint stress, can induce depressive-like behaviour^17,18^. It also has been shown that exposure to psychosocial stress leads to a much wider range of depressive-like behaviours and more pronounced increases in pro-inflammatory cytokine profiles than exposure to predominately physical stress and/or UCMS^4,17^. Although these lines of research have shed some light on the impact of different types of stress, only one of these studies directly compared different stress paradigms in the same study and, moreover, was limited in the biological domains assessed, having focused specifically on the neuroendocrine system^4^. Therefore, a more comprehensive evaluation is needed, one that focuses on multiple behaviours and biological outcomes resembling the complexity of MDD.

Therefore, with this in mind, we aimed to investigate the effect of two well-established stressors, social isolation^1,11^ and repeated injections^19–21^, both for 9 weeks in young adult male animals. Although depression can develop at any age between early childhood and older adulthood, both national and cross-national epidemiological studies report that the first onset of depression most frequently occurs in the 20s to early 30s^22–24^, and thus the animal equivalent of a young adult was purposely selected for study. We measured anxiety- and/or depressive-like behaviour and corticosterone responsivity, systemic inflammation, neuroinflammation and hippocampal neurogenesis—biological outcomes all associated with MDD^25^. We wanted to establish whether we can discriminate between the effects of repeated injection and permanent social isolation with respect to the behavioural and biological outcomes, and whether combining these stressors alters the severity of the associated behavioural and/or biological outcomes.

## Materials and methods

### Animals

Experiments were conducted with male BALB/cAnNCrl mice (*n* = 50), aged 6–7 weeks, weighing 22–26 g, obtained from Charles River Laboratories (Margate, Kent, UK). Animals were housed under standard conditions (19–22 °C, humidity 55%, 12 : 12 h light : dark cycle with lights on at 07:00, food and water ad libitum) and had a 1-week period, prior to stress exposure, to acclimatize to the Biological Services Unit and experimenter. During habituation, all animals were housed in sibling pairs. All housing and experimental procedures were carried out in compliance with the local ethical review panel of King’s College London and the UK Home Office Animals Scientific Procedures Act 1986. For the rationale behind the chosen strain, sex and age of the mice used, see Supplementary Material.

### Stress exposure paradigms

**Fig. 1:**
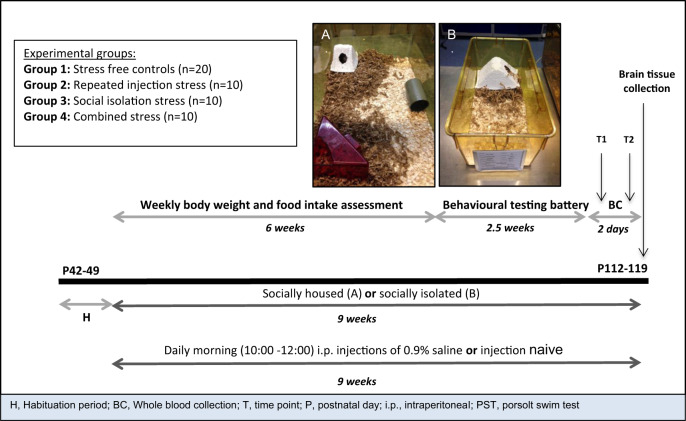
Schematic representation of the experimental procedure. Mice were exposed to one of four experimental conditions after an initial 1-week habituation period: (i) stress-free control group (socially-housed in pairs and injection naive—Group 1); (ii) repeated injection stress (socially-housed in pairs and repeatedly injected—Group 2); (iii) social isolation stress (permanent social isolation and injection naive—Group 3); and (iv) combined stress (permanent isolation and repeatedly injected—Group 4). Behavioural testing began after 6 weeks of treatment and was carried out under stringent environmental control, using standardized test procedures, between 09:00 and 15:00, unless otherwise stated. Blood was collected 24 h before and 30 min after acute stress exposure, i.e., the Porsolt Swim Test, in the final week of behavioural testing. Animals were culled 24 h later and brain tissue extracted. **a** Housing for sibling pair-housed animals (Groups 1 and 2): large techniplast cages (45 × 32 × 20 cm) were set up with sawdust, a red house, white house, nesting material and a plastic enrichment tube. Larger cages with more nesting material, shelter and enrichment were deemed necessary to reduce aggression between sibling pair-housed animals. **b** Housing for singly-housed animals (Groups 3 and 4): small tecniplast cages (32 × 16 × 14 cm) were set up with sawdust, a white house and nesting material. Note: a larger sample size was used for control animals (*n* = 20) given the risk of group housing such an aggressive strain (Deacon, 2006; Charles Rivers Laboratories International, Inc., 2012; www.criver.com).

Mice were exposed to one of three stress treatments for a 9-week duration. Each treatment comprised one or two distinct stressors that was either in the form of repeated injection, which has been previously shown to differentially alter stress responses in BALB/C mice^20^ and affective outcomes in outbred rats with high and low emotional reactivity^21^, or permanent social isolation, which has consistently been associated with depressive-like phenotypes^8,9,11^. In brief, three groups of mice were exposed either to repeated injection only (Group 2), permanent social isolation only (Group 3) or a combination of both stressors (hereafter referred to as combined stress; Group 4), whereas one group remained stress free (Group 1). Group sample sizes were based on numbers typically used in animal research using chronic stress models^1,10^. Animals, either singly or as a sibling pair, were randomly assigned by a chance procedure to their respective groups after the initial habituation period. For all forms of assessment, the experimenter was blinded to group status and all animals were handled regularly. Figure 1 defines in more detail the experimental groups and depicts the experimental timeline and housing conditions.

### Intraperitoneal injection procedure

Intraperitoneal injections were carried out according to recommended guidelines and as previously described^26,27^. Briefly, animals were scruffed and injected with saline volumes of 10 ml/kg body weight administered between 10:00 and 12:00 daily. Injection naive animals were handled but not injected. For details on the injection procedure, see Supplementary Material.

### Physical health assessment

Body weight and food intake was measured weekly during the first 6 weeks of stress exposure (Fig. 1). For details on these measures, see Supplementary Material.

### Behavioural assessment

Anxiety-like behaviour was assessed using the Open-Field Test (OFT)^28^ and Novelty Suppressed Feeding Test (NSFT)^29^, whereas depressive-like behaviour was measured using the Sucrose Preference Test (SPT)^30^ and Porsolt Swim Test (PST)^31^—all as previously described. Behavioural testing was carried out after 6 weeks of stress exposure (Fig. 1). For details on the general conditions on all behavioural testing and the methods for all behavioural assays, see Supplementary Material.

### Biochemical assessment

#### Blood sampling, processing and storage

Whole blood, from a tail cut, was collected between 09:00 and 12:30, both 24 h before and 30 min after acute stress exposure, i.e., the PST, at the end of behavioural testing. Samples were centrifuged at 3000 r.p.m. for 10 min at 4 °C and plasma was removed and stored at −80 °C. For details on blood sampling, see Supplementary Material.

#### Corticosterone assessment and stress reactivity

Corticosterone levels were measured in duplicates from plasma samples collected at both blood collection points using commercially available enzyme-linked immunosorbent assay (ELISA) kits (Enzo Life Sciences, Switzerland), according to the manufacturer’s instructions. Absorbance values were converted into concentrations using cubic spline four parameter logistics.

#### Cytokine assessment

Plasma cytokine levels were determined using the multiplex screening assay based on magnetic Luminex® xMAP® technology as previously described^32^. Using a custom-made mouse premixed multi-analyte magnetic luminex screening assay (R&D Systems, Minneapolis, USA), levels of interleukin (IL)-1β, IL2, IL4, IL6, IL10, IL12, C-reactive protein, vascular endothelial growth factor (VEGF), insulin-like growth factor and tumour necrosis factor (TNF)-α, in blood plasma collected at both blood collection points, were measured according to the manufacturer’s instructions. Median fluorescence intensity values were converted into concentrations using cubic spline five parameter logistics. See Supplementary Material for details on the ELISA and Luminex protocols, inter- and intra-assay variability, and kit sensitivity.

### Histological assessment

#### Brain tissue collection and sectioning

Animals were transcardially perfused and brains quickly extracted as previously described^33^. All brain tissue was coronally sectioned at a thickness of 40 μm using a Leitz freezing microtome (Microm HM430, Carl Zeiss Ltd, Cambridge, UK) as previously described^34,35^. For details on brain tissue collection, sectioning and storage, see Supplementary Material.

#### Free-floating immunoperoxidase labelling

Proliferative cells, immature neurons, microglia, astrocytes and mature astrocytes were visualized using Ki67, doublecortin (DCX), ionized calcium-binding adapter molecule-1 (Iba1), glial fibrillary acidic protein (GFAP) and s100β, respectively, using free-floating immunohistochemistry as described previously^36^. For protocol details, antibodies used and representative images, see Supplementary Material and Supplementary Fig. 1.

#### Stereological analyses of volume and immunopositive cell density

The cell density of immunopositive Ki67, DCX, Iba1, GFAP and s100β cells in the prefrontal cortex (PFC) and/or the dentate gyrus (DG) of the hippocampus were estimated by stereological analysis with StereoInvestigator software (MBF Bioscience, Williston, VT) using the optical fractionator module as previously described^37^. For details on the stereological methods, see Supplementary Material.

#### Processing of Iba1 and GFAP-positive images

Whole-slide digital images were provided by the UCL IQpath slide scanning service, using a Leica SCN400F scanner. Representative images of the hippocampus and the PFC were captured with Aperio Imagescope software v12.2.2 (Leica Biosystems, UK) at ×2 magnification.

#### Threshold analyses of immunoreactivity for Iba1- and GFAP-positive cells

To quantify and compare relative levels of immunoreactivity for Iba1- and GFAP-positive cell staining in the DG and PFC, thresholding analyses using ImageJ software v1.51^38^ were performed as described previously^39,40^. See Supplementary Material for details of the thresholding methodology.

#### Morphometric analyses of Iba1-positive cells

Using ImageJ software v1.51^38^ sholl and skeleton analyses were performed as previously described^39–41^. For all analyses, 18 Iba1 cells were randomly selected across three hippocampal sections per mouse. Three mice per experimental group were used for morphological quantification, equating to a total of 54 Iba1 cells per group. For details of the morphometric methodology, see Supplementary Material.

#### Immunofluorescent double labelling of GFAP and SOX2

Given that GFAP is also a marker for neural stem cells^42^, to determine whether changes observed in GFAP immunoreactivity were related specifically to astrocytes, sections were fluorescently double-labelled to determine the extent of co-localization of GFAP with SOX2—a neural stem cell specific marker^43^. Immunofluorescent double labelling was carried out as previously described^44^. For details on the protocol and representative images, see Supplementary Material and Supplementary Fig. 2.

#### Immunofluorescent image processing

Images of double-labelled sections were obtained using a Leica SP5 confocal microscope. Specifically, an objective of ×63 (oil immersion, NA 1.42) was used for each image captured and wavelength laser lines of 405 nm (diode laser), 488 nm (argon laser) and 594 nm (HeNe laser) were used. All images were acquired as confocal stacks of ten images separated by a 0.64 μm *z*-axis step size. Each image was taken at a resolution of 1024 × 1024 pixels, with the picture dwell time set to 3.36, giving a rate of 0.185 *z*-planes per second, and each frame was averaged four times to reduce signal noise. Additional settings of gain, offset and pinhole size were optimized prior to imaging and were held constant for all images at 600 V, 800 V and 1.0 airy unit, respectively.

#### Immunofluorescent cell quantification

For each confocal stack, the percentage of double-labelled cells for GFAP and SOX2 in the DG of the hippocampus was determined using methods as outlined previously^45^. Briefly, 100 GFAP-positive cells per mouse were counted and the percentage of radial glial cells (GFAP+/SOX2+) and astrocytes (GFAP+/SOX2−) were calculated. For each mouse, cells were counted from a total of 12 acquired *z*-stacks across 6 hippocampal sections.

#### Immature neuron classification based on DCX morphology

DCX-positive cell morphology in the DG was visually classified as previously described^46^. In brief, DCX-positive cells were classified according to four neuroblast subtypes based on their level of maturation, and the cell density for each neuroblast cell type was determined using stereological analyses as aforementioned. For details on the classification process, see Supplementary Material and Supplementary Fig. 3.

### Statistical analyses

Statistical comparisons were conducted in IBM SPSS Statistics v.23 (IBM Ltd, Portsmouth, UK) and consisted of independent samples *t*-tests, one-way or two-way analyses of variance (ANOVA), repeated-measures two-way ANOVA, three-way factorial ANOVA, repeated-measures generalized linear mixed modelling, Mann–Whitney, or Kruskal-Wallis, followed by Bonferroni or Dunn’s post hoc analyses where appropriate. All data were assessed for normality using probability–probability plots and the Kolmogorov–Smirnov test, and for homogeneity of variance using the Levene’s test. For data that did not conform to normality and/or homoscedasticity non-parametric statistical tests were applied. All tests carried out were two-sided and the alpha criterion used was *p* < 0.05. Data are represented as the mean and SEM or the median and interquartile range. All animals were included in all analyses. For a more detailed description of the analytical approach, see Supplementary Material.

## Results

### All types of chronic stress induce anxiety-like behaviour, but only social isolation increases depressive-like behaviour

**Fig. 2:**
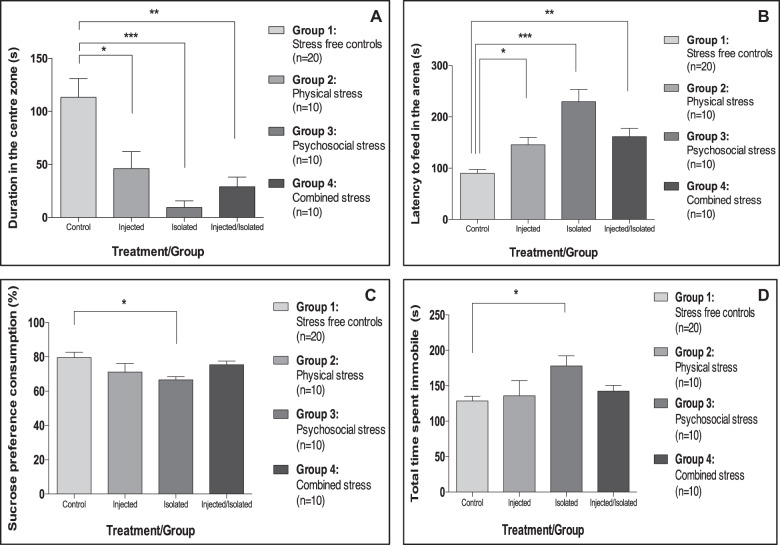
Effect(s) of repeated injection, social isolation and combined stress on anxiety- and depressive-like behaviour. **a** Mean (±SEM) centre duration in the OFT (F[3,46] = 8.9, *p* < 0.001). **b** Median (±IQR) latency to feed in the NSFT (K[4,50] = 21.6, *p* < 0.001). **c** Mean (±SEM) % sucrose preference consumption in the SPT (F[3,46] = 3.0, *p* = 0.04). **d** Mean (±SEM) immobility duration in the PST (F[3,46] = 2.9, *p* = 0.045). *p < 0.05; **p < 0.01; ***p < 0.001 relative to control (adjusted *p*-values). Analyses: one-way ANOVA or Kruskal-Wallis with Bonferroni or Dunn’s post hoc correction, respectively.

We first assessed the behavioural effects associated with chronic stress exposure and found that all stress-exposed mice, irrespective of the type of stress, exhibited significantly increased anxiety-like behaviour in the OFT (Fig. 2a) relative to control animals, as shown by significantly less time in the centre of the OFT arena (−59% in repeatedly injected animals, −92% in the socially isolated mice and −74% in animals exposed to combined stress spent (Fig. 2a)).

Moreover, all groups of stress-exposed mice displayed an increased latency to feed in the NSFT relative to control animals (Fig. 2b) but due to the significant increases in anxiety also observed in these mice in the OFT, the NSFT was heavily confounded by anxiety, as the task is run in a novel arena. Therefore, it was not possible to use this test to specifically assess anhedonia.

Interestingly, only mice exposed to permanent social isolation exhibited signs of significant depressive-like behaviour when compared with controls (Fig. 2c, d), as shown by increased anhedonic-like behaviour (exposed animals consuming 16% less sucrose in the SPT; Fig. 2c), and behavioural despair (exposed animals spending 38% more time immobile in the PST; Fig. 2d).

Notably, behaviour in the OFT and NSFT was not confounded by locomotor activity (Supplementary Fig. 4A, B), and the observed differences in the SPT were not attributed to either total liquid consumption (Supplementary Fig. 4D) or food consumption (Supplementary Fig. 4E). Moreover, the observed differences in the behavioural assays were not confounded by olfactory ability (Supplementary Fig. 4C) or by social dominance (Supplementary Table 1).

For further detail on all other behavioural readouts, see Supplementary Fig. 5 and for a full summary of all the behavioural changes associated with the three chronic stress exposures, see Supplementary Table 2.

### Chronic stress does not alter baseline corticosterone levels after stress exposure, but the type of stress differentially alters corticosterone responsivity

**Fig. 3:**
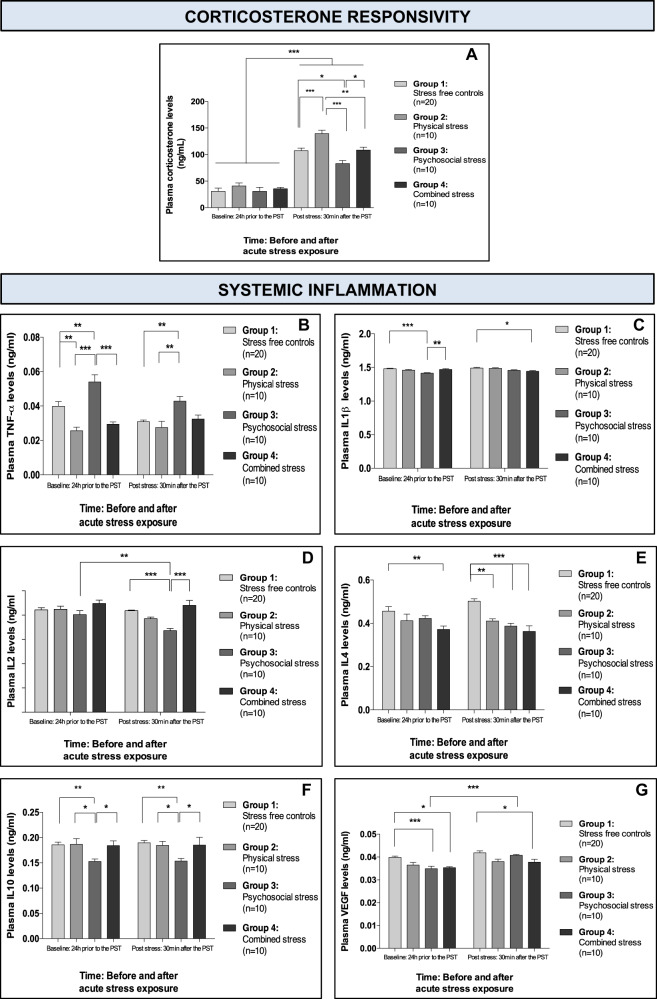
Effect(s) of repeated injection, social isolation and combined stress on corticosterone responsivity and systemic inflammation. **a** Mean (±SEM) plasma corticosterone levels before and after acute stress exposure. **b** Mean (±SEM) plasma TNF-α levels before and after acute stress exposure. **c** Mean (±SEM) plasma IL1β levels before and after acute stress exposure. **d** Mean (±SEM) plasma IL2 levels before and after acute stress exposure. **e** Mean (±SEM) plasma IL4 levels before and after acute stress exposure. **f** Mean (±SEM) plasma IL10 levels before and after acute stress exposure. **g** Mean (±SEM) plasma VEGF levels before and after acute stress exposure. *p < 0.05; **p < 0.01; ***p < 0.001 (adjusted *p*-values). Analyses: repeated-measures two-way ANOVA with Bonferroni post hoc comparison.

We next sought to uncover some of the biological alterations associated with these behavioural perturbations in stressed-exposed mice, focusing first on peripheral changes related to HPA axis functioning. We found no significant differences in baseline corticosterone levels across experimental groups after chronic stress exposure (i.e., 24 h before acute stress), and all mice showed a 256% average increase in plasma corticosterone in response to the PST (Fig. 3a). Interestingly, corticosterone levels 30 min after the acute stress did significantly differ between experimental groups (Fig. 3a). On average, levels increased by threefold after the PST; however, mice exposed to repeated injection had a 41% larger increase in corticosterone levels compared with controls, whereas socially isolated mice had a 22% smaller increase in corticosterone levels compared with controls, and mice exposed to combined stress had a stress response similar to that of controls (Fig. 3a).

For a detailed summary of all the peripheral corticosterone data for each of the three chronic stress exposures, see Supplementary Table 3.

### Repeated injection decreases TNF-α and IL4; social isolation increases TNF-α, but decreases IL1β, IL2, IL4, IL10 and VEGF; and injection and social isolation combined decreases IL1β, IL4 and VEGF

We next examined the impact of chronic stress exposure on peripheral inflammatory profiles, both at baseline (i.e., 24 h before the PST) and after the acute stress. Interestingly, we found that there was very little pre vs. post effect of the acute stress on any of the cytokines (Fig. 3b–g). Specifically, only socially isolated mice exhibited significant differences in cytokine plasma levels post-PST relative to baseline, with a decrease in IL2 and an increase in VEGF levels in response to acute stress.

In terms of between-group comparisons vs. control mice, mice exposed to repeated injection had significantly lower baseline TNF-α and post-PST IL4 levels (Fig. 3b, e). Conversely, socially isolated mice had significantly higher baseline and post-PST TNF*-*α levels, lower baseline and post-PST IL10 levels, together with lower levels of baseline IL1β and VEGF, and post-PST IL2 and IL4 (Fig. 3b–g). Mice exposed to combined stress had consistently lower IL4 and VEGF at baseline and post-PST, and lower IL1β levels post-PST (Fig. 3c, e, g).

Intriguingly, no significant differences in IL6 or IL12 were found between (or within) experimental groups, pre- and/or post-PST (Supplementary Fig.6 A, B). For a detailed summary of all the inflammatory data for each of the three chronic stress exposures see Supplementary Table 3.

It is also noteworthy that none of the reported inflammatory changes were artifacts of social hierarchy (Supplementary Table 4).

Given that glial cells play an important role in immune system functioning^47^, we next examined both microglial and astrocyte biology in both the hippocampus and PFC—two particularly stress-sensitive regions^48^.

### Repeated injection increases Iba1-positive cell density and promotes a dystrophic cell morphology in the ventral DG, whereas social isolation decreases Iba1-positive cell density and induces a ramified cell morphology in the dorsal DG

**Fig. 4:**
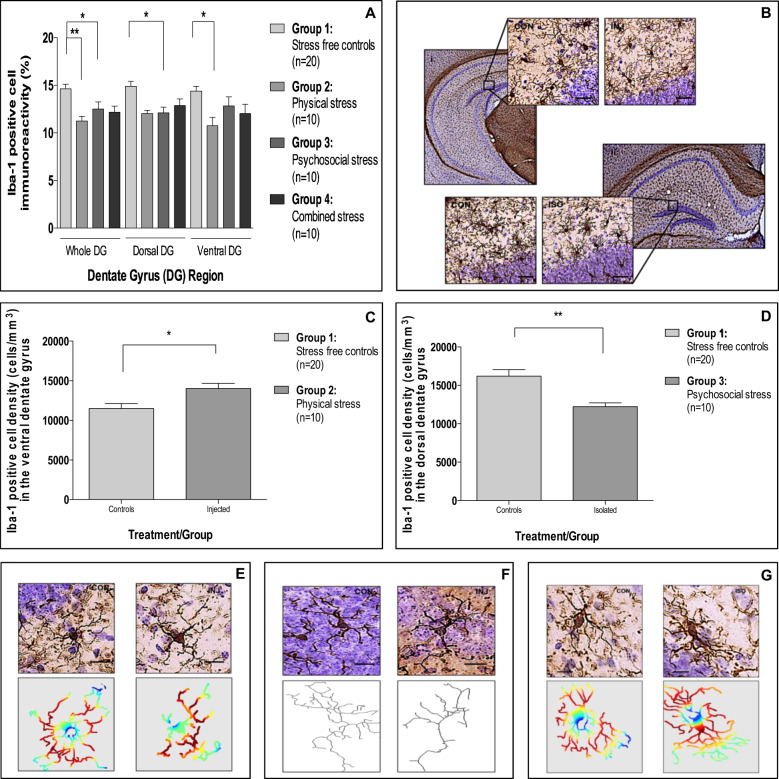
Effect(s) of repeated injection, social isolation and combined stress on microglial biology. **a** Mean (±SEM) Iba1-positive cell immunoreactivity in the dentate gyrus (Effect of exposure: F[3,138] = 5.7, *p* = 0.002; Effect of region: F[2,138] = 1.2, *p* = 0.29; Interaction: F[3,138] = 0.9, *p* = 0.45). **b** Representative photomicrographs of the ventral (i) and dorsal (ii) dentate gyrus stained for Iba1 for repeatedly injected and socially isolated mice, respectively, all relative to controls. Images captured at ×2 and ×20 magnification, scale bar = 50 μm. **c** mean (±SEM) Iba1-positive cell density in the ventral dentate gyrus for repeatedly injected mice (*t* [28] = 2.7, *p* = 0.02). **d** Mean (±SEM) Iba1-positive cell density in the dorsal dentate gyrus for socially isolated mice (*t* [28] = 4.2, *p* = 0.001). **e** Representative photomicrographs and associated sholl masks of Iba1 cells in the ventral dentate gyrus of repeatedly injected and control animals. **f** Representative photomicrographs and associated skeletons of Iba1 cells in the ventral dentate gyrus of repeatedly injected and control animals. **g** Representative photomicrographs and associated sholl masks of Iba1 cells in the dorsal dentate gyrus of socially isolated and control animals. Images **e**–**g** were taken at ×40 magnification, scale bar = 40 μm. In Sholl masks, red denotes a greater degree of branching complexity, whereas blue denotes the lowest degree of branching complexity. *p < 0.05; **p < 0.01; ***p < 0.001 (adjusted *p*-values). Analyses: two-way ANOVA with Bonferroni post hoc comparison or Independent samples *t*-test.

Looking at microglial biology first, we found that exposure to repeated injections and social isolation reduced Iba1 immunoreactivity in the DG of the hippocampus, with a similar pattern, but not reaching statistical significance, for the combined stress (Fig. 4a, b). These effects in the DG had specific regional selectivity, with decreases that were significant in the ventral DG for the repeatedly injected mice (−19%) and in the dorsal DG for the socially isolated mice (−25%) (Fig. 4a, b). Interestingly, none of the conditions affected Iba1-positive cell immunoreactivity in the PFC (Supplementary Fig. 7A, B).

To further understand the relevance of these changes in Iba1 immunoreactivity, we next assessed cell density and morphology in these specific regions of the DG. Interestingly, despite both repeated injections and social isolation showing a decrease in Iba1 immunoreactivity, repeatedly injected mice had a 22% increase in Iba1 cell density in the ventral DG (Fig. 4c), while socially isolated mice had a 24% decrease in Iba1 cell density in the dorsal DG (Fig. 4d).

Moreover, although injected mice had an increased Iba1 cell density in the ventral DG, the morphological characteristics of these cells were altered, such that they assumed a more dystrophic morphology, potentially explaining the decreased Iba1 immunoreactivity data for this group (Fig. 4e, f and Supplementary Table 5). In direct contrast, Iba1 cells of socially isolated animals assumed a more ramified morphology, with no difference in the amount of space each cell occupies observed (Fig. 4g and Supplementary Table 5). It is also noteworthy that cell soma size and average process length was not different between experimental groups (Supplementary Table 5).

### Social isolation increases GFAP-positive cell immunoreactivity and s100β-positive cell density in the DG

**Fig. 5:**
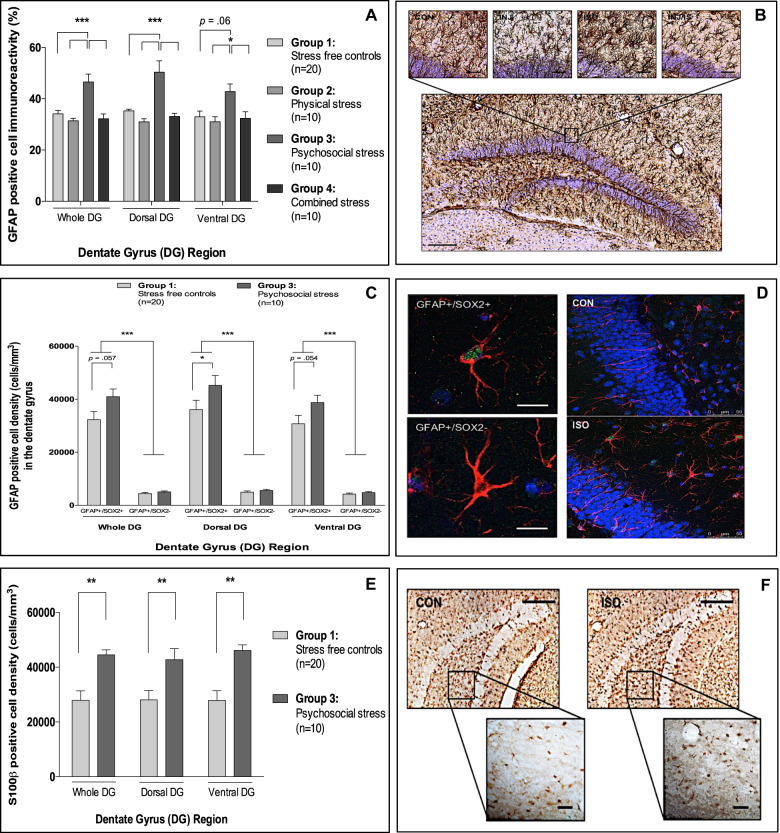
Effect(s) of repeated injection, social isolation and combined stress on astrocyte biology. **a** Mean (±SEM) GFAP-positive cell immunoreactivity in the dentate gyrus (Effect of exposure: F[3,138] = 15.7, *p* < 0.001; Effect of region: F[2,138] = 2.1, *p* = 0.15; Interaction: F[3,138] = 0.9, *p* = 0.45). **b** Representative photomicrograph of the dorsal dentate gyrus from a representative control stained for GFAP (×10 magnification; scale bar = 100 μm) and close-up representative photomicrographs for each experimental group (×20 magnification; scale bar = 50 μm). **c** mean (±SEM) GFAP-positive cell density in the dentate gyrus stratified by GFAP cell type and dentate gyrus region (Effect of exposure (**a**): F[1,168] = 6.9, *p* = 0.01; Effect of region (**b**): F[2,168] = 3.6, *p* = 0.06; Effect of cell type (**c**): F[1,168] = 350.8, *p* < 0.001. Interactions: (**a** × **b**) F[1,168] = 0.03, *p* = 0.87; (**a** × **c**) F[1,168] = 5.2, *p* = 0.03; (**b** × **c**) F[1,168] = 2.2, *p* = .15; (**a** × **b** × **c**) F[1,168] = 0.02, *p* = 0.88). **d** Representative photomicrographs of a radial glial cell (GFAP+/SOX2+) and astrocyte (GFAP+/SOX2−) taken from socially isolated animals (×40 magnification; scale bar = 40 μm) and representative maximum intensity 2D *z*-stack projections of ten confocal images taken from socially isolated and control animals (×20 magnification; scale bar = 50 μm). **e** Mean (±SEM) s100β-positive cell density in the dentate gyrus (Effect of exposure: F[1,84] = 24.0, *p* < 0.001; Effect of region: F[2,84] = 0.2, *p* = 0.64; Interaction: F[1,84] = 0.3, *p* = 0.61). **f** Representative photomicrographs of s100β-positive cells in the ventral dentate gyrus of controls and socially isolated mice (×40 magnification; scale bar = 50 μm). *p < 0.05; **p < 0.01; ***p < 0.001 (adjusted *p*-values). Analyses: two-way ANOVA with Bonferroni post hoc comparison or three-way factorial ANOVA with Bonferroni post hoc comparison.

In addition to investigating the impact of chronic stress on microglial biology, we also examined astrocyte biology in both the DG and PFC. Interestingly, only social isolation stress increased GFAP immunoreactivity in the DG relative to all other groups (47%)—a change not DG region specific (Fig. 5a, b)—and similar to Iba1 data, there were no differences in GFAP immunoreactivity in the PFC between experimental groups (Supplementary Fig. 7C, D).

As GFAP is also expressed in neural progenitor cells, immunofluorescent double labelling was subsequently used to further characterize these cells in socially isolated and control animals. Interestingly, our data revealed that all mice irrespective of exposure had a radial glia to astrocyte cell ratio of 9 : 1 (Supplementary Fig. 8).

Given that only ~10% of GFAP-positive cells in the DG were astrocytes (i.e., controls: 12%; isolated: 11%), stereological cell density estimates were next obtained to determine whether the observed differences in GFAP immunoreactivity were related to one or both GFAP-positive cell types. Specifically, we found that social isolation significantly increased the number of radial glial cells only in both the dorsal (25%) and ventral DG (26%) (Fig. 5c, d).

As only a small proportion of GFAP-positive cells were A1 astrocytes in the context of our work, s100β-positive cell density—typically a marker of A2 astrocytes—was next examined. Similar to GFAP, we found that social isolation significantly increased s100β-positive cell density in both the dorsal (52%) and ventral DG (65%) (Fig. 5e, f).

### Chronic stress does not alter neuronal proliferation but does alter neuronal differentiation

**Fig. 6:**
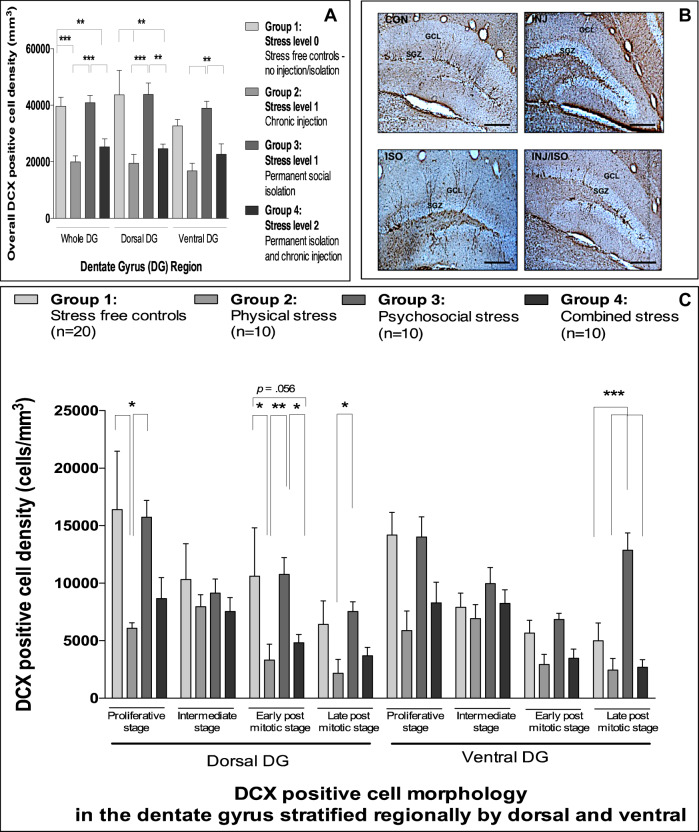
Effect(s) of repeated injection, social isolation and combined stress on neurogenesis. **a** Mean (±SEM) DCX-positive cell density in the dentate gyrus (Effect of exposure: F[3,138] = 28.9; *p* < 0.001; Effect of region: F[2,138] = 2.0; *p* = 0.14; Interaction: F[6,138] = 0.3; *p* = 0.94). **b** Representative photomicrographs of the dorsal dentate gyrus for all experimental groups stained for DCX (×10 magnification; scale bar = 100 μm). **c** Mean (±SEM) DCX-positive cell morphology in the dentate gyrus (Effect of exposure (**a**): F[3,368] = 26.9, *p* < 0.001; Effect of region (**b**): F[1,368] = 2.2, *p* = 0.14; Effect of cell type (**c**): F[3,368] = 20.3, *p* < 0.001; Interactions: (**a** × **b**) F[3,368] = 1.1, *p* = 0.36; (**a** × **c**) F[9,368] = 2.2, *p* = 0.02; (**b** × **c**); F[3,368] = 1.5, *p* = 0.22; (**a** × **b** × **c**); F[9,368] = 0.8, *p* = 0.59). *p < 0.05; **p < 0.01; ***p < 0.001 (adjusted *p*-values). Analyses: two-way ANOVA with Bonferroni post hoc comparison or three-way factorial ANOVA with Bonferroni post hoc comparison.

Finally, given the associations between hippocampal neurogenesis, depression, stress and inflammation^49^, we investigated the impact of the three chronic stress paradigms on both proliferation and differentiation in the DG. We found that chronic stress exposure did not alter hippocampal volume (Supplementary Fig. 9A) or Ki67 cell density in the DG (Supplementary Fig. 9B, C) for any of the exposure groups. However, mice exposed to repeated injection, or the combined stress, did show a decrease in overall DCX-positive cell density (−48%) in the dorsal DG relative to controls (Fig. 6a, b). Surprisingly, socially isolated mice had an overall DCX-positive cell density similar to controls.

To gain a better understanding of whether these reductions in DCX cell density were related to all DCX cell types, or whether they were specific to a particular stage of DCX maturation, we classified all DCX-positive cells into one of four morphology types. Compared with control mice, we found a decrease in all neuroblast cell types (−66%) and in early post mitotic cells (−55%) in the dorsal DG for repeatedly injected mice and mice exposed to combined stress, respectively. Interestingly, we also found an increase in post mitotic stage neuroblasts (71%), in the ventral DG, in socially isolated animals. No differences in intermediate stage neuroblast cell density were found for any of the exposure groups (Fig. 6c).

None of the reported brain-related changes were artifacts of social hierarchy (Supplementary Tables 6 and 7), and for a full summary of all these changes, see Supplementary Table 8.

## Discussion

For the first time, we show how exposure to different types of chronic stressors elicits distinct behavioural and biological phenotypes in both the periphery and the brain. Overall, our results clearly demonstrate that the type of chronic stress exposure can indeed matter. Our most interesting finding is that distinct types of chronic stress differentially alter hippocampal neuroinflammatory and neurogenic profiles, which may be the basis by which the different behavioural phenotypes ultimately manifest. Based on our data, we believe that the neurogenic profiles observed are a functional consequence of the neuroinflammatory changes associated with each stress exposure, given that microglia and astrocytes play an important role in maintaining synaptic integrity^50^. Moreover, given that glucocorticoids and peripheral inflammation are the two most well-known pathways through which stress can affect microglia/astrocyte structure and functioning^51–53^, we believe that altered HPA axis activity and cytokines levels, also found in our models, are the key systems involved in altering microglia/astrocyte/neurogenesis and ultimately behaviour.

Our data suggest that repeated injections promotes HPA axis hyperactivity (possibly related to the anxious phenotype), as shown by increased corticosterone reactivity to stress, together with lower TNFα and IL4 levels, indicating a hypercortisolemia-associated inhibition of the peripheral immune system^54^. Moreover, HPA axis hyperactivity can lead to increased microglial activation^53^, which is also seen in repeatedly injected mice, as indicated by the increased Iba1 cell density, reflecting microglia rapidly proliferating once activated^55^, and Iba1 cell morphology resembling reactive microglia^51^, given that these cells occupy less overall space, possess a shorter maximum process length, and have fewer processes and reduced branching ramification. Interestingly, previous research demonstrates that activated microglia assume a phagocytotic role and contribute to the apoptosis of newborn neurons^56^, and consistent with this we observe a concomitant decrease in neuronal differentiation in these same animals. Given that there is no change in Ki67, which is predominately expressed during the earlier critical neurogenic period, and there is a decrease in more mature neuroblasts (but not in the intermediate stage), we can conclude that the majority of apoptosis is likely occurring during the later 1–3 week window, a critical period for newborn cell survival^52^. However, it is notable that we also see a significant reduction in proliferative stage neuroblasts, which supports that some cell death also occurs in the earlier critical period, although as apoptosis was not quantified in our study, this requires validation.

Contrary to repeated injection, permanent social isolation promotes HPA axis hypoactivity as shown by decreased corticosterone reactivity to stress, together with higher TNFα (i.e., an exact mirror image of the repeated injections), together with decreased IL10, IL1β, VEGF and IL4. Importantly, IL10 is an important mediator for suppressing the immune response and directly inhibits the proliferation and production of TNFα^57^. Furthermore, increased TNFα and decreased IL10 are both strongly associated with depressive-like behaviour and clinical depression^58–60^—a behavioural phenotype found only in our socially isolated animals.

As with repeated injection, we believe that these different changes in HPA axis/immune system activity promote the microglial/astrocyte-associated abnormalities observed in socially isolated mice^61^. For example, the reduced levels of corticosterone and the increased levels of pro-inflammatory cytokines promote microglial overactivation^44,62^, which can lead to increased microglial apoptosis^63^ and hyper-ramification^62^, which we observe in our socially isolated animals, as indicated by a decrease in Iba1 cell density and an increase in the internal complexity of these cells. Moreover, we find an activation of neuroprotective A2 astrocytes, as shown by the increased GFAP- and s100β-positive cell density, the latter of which is specifically associated with A2 astrocytes^64^, findings that have already been described in association with stress^65–67^, including in our own in vitro work mimicking ‘stress in a dish’ with low concentration of glucocorticoids^68^.

As microglia and astrocytes are vital for synaptogenesis and/or synaptic integrity/maintenance^56,69–71^, and that their structure is intrinsically linked to their function^72^, we speculate that the changes in neurogenesis following social isolation are functionally linked to the observed neuroinflammatory changes. Indeed, in the socially stressed animals, we see a specific increase in mature neuroblasts, suggesting an overall impairment in synaptic pruning and/or apoptosis inhibition. Given that we observe a reduced number of Iba1 cells with an altered morphology, and an increase in A2 astrocyte activation, we believe that these glial cells are not able to adequately perform their function of synaptic pruning and/or overcompensate their neuroprotective-associated functions^66^. Further evidence to support these functional abnormalities comes from our finding that VEGF is reduced in these animals, since VEGF regulates synaptic pruning and synapse formation^73^, as well as neurogenesis and microglia^74^, and that astrocytes can directly secrete VEGF^75,76^.

Interestingly, we not only find a wider regional specificity of chronic stress exposure for both neuroinflammatory and neurogenic profiles between the PFC and the hippocampus, but also regional specificity within the DG. Although, the finding that regional differences exist is not novel^48,77^, finding no impact of chronic stress on the PFC is contrary to previous research^48,62,78,79^. However, the type and duration of stress exposure can have important implications on microglia and/or astrocyte biology^36^, and we cannot out rule that changes in the PFC may have occurred earlier than measured in this study.

Although the basis for the regional specificity within the DG is unclear, one potential explanation could relate to the microglial response, which might occur at a different rate, or endure for different periods of time, in response to each of the stress exposures. Moreover, given that our differentially stressed animals respond so distinctively to acute stress, a differentially altered microglial response represents a parsimonious explanation and especially when glucocorticoids are well-known modulators of microglial function^80^.

It is also notable that the ventral hippocampus (relative to the dorsal) responds entirely differently to glucocorticoids, such that it has a reduced firing frequency accommodation and more depolarization-associated spikes^81^. This differential response may allow for a longer window of acquisition when activated^82^ and given the prominent role of the ventral hippocampus in inhibiting the HPA axis, raises the possibility that the differential actions of glucocorticoids on synaptic function may be relevant to stress regulation. In our work, this could potentially contribute to the regional specificity observed for both neuroinflammatory and neurogenic profiles in stress-exposed animals.

Regarding a broader interpretation of the functional significance of the neurogenesis-related findings, it is well-known that the dorsal DG plays a role in memory and cognition, while the ventral DG controls stress responsivity and emotional processing^83^. Therefore, it is not entirely surprising that DCX in the ventral region of the DG is specifically altered in the context of permanent social isolation, which promotes a robust depressive-like phenotype, and a decrease in stress responsivity. Moreover, these preferential effects on the ventral DG have previously been reported^84–88^.

Although our data on social isolation aligns with the functional relevance of the ventral DG, it is unknown whether this applies to repeated injection and its associated changes in the dorsal DG when cognitive behaviour was not measured. However, it is noteworthy that anxiety-like behaviour and cognitive impairment are closely associated in the context of chronic stress^89–91^ and future research should therefore measure cognitive functioning to extend upon our findings.

Unlike repeated injection and/or social isolation, we find that combined exposure to these stressors surprisingly does not induce depressive-like behaviour, alter microglial/astrocyte biology, or alter HPA axis activity, contrary to previous research^36,48,92,93^. Perhaps the most powerful conclusion of our study is that there appears to be no synergistic, potentiating effect in combining such different stressors, and in fact the two specific phenotypes of hypercortisolemia/reduced inflammation (repeated injection) and hypocortisolemia/increased inflammation (social isolation) seem to cancel each other out, leading to no differences in these biological systems. However, we do observe a specific decrease in overall neuronal differentiation in these stressed animals, for which altered cell death and/or cell proliferation will still ultimately account for these changes. Moreover, these animals also exhibit specific decreases in IL4, IL1β and VEGF, and given that VEGF is an important pro-neurogenic growth factor^94,95^, and that cytokines can independently modulate neurogenesis^96,97^, these changes in immune system functioning could account for the observed decrease in neurogenesis. Pertinently, a lack of IL4 specifically has previously been shown to promote anxiety- not depressive-like behaviour^98^—a behavioural outcome also observed in our combined stress-exposed mice. Therefore, the observed decrease in IL4, together with reduced neurogenesis, could ultimately contribute to the aberrant behavioural profile of these animals.

Although we clearly demonstrate that differential types of stress can indeed promote unique phenotypes, we believe that nociception could potentially explain these differential outcomes, especially when repeated injection initiates an acute pain response, whereas social isolation does not. The immune system, HPA axis and nociception are all intrinsically linked^99,100^, and therefore this could account for some of the observed biological and behavioural phenotypes associated with the two types of stress. For example, we specifically see reduced TNFα in repeatedly injected animals, and TNFα is an important inflammatory mediator of pain^101^, the inhibition of which has been shown to alter pain perception^102^. Thus, the observed biological changes associated with our repeated injection paradigm could be a reflection of the pain response and/or a change in nociception sensitivity.

Pertinently, chronic pain has been consistently associated with anxiety^103**–**105^—a behavioural outcome observed for our repeatedly injected animals. Furthermore, preclinical studies using electric foot shock paradigms, which also elicit pain, likewise report an increase in anxiety-like behaviour^106,107^, and clinical studies using repeated pain stimulation show that, despite pain habituation, exposed individuals report increased anxiety^108^. Interestingly, previous research shows how repeated injection differentially alters affective outcomes in rats with high and low emotionality, with low responders showing no change in depressive-like states^21^. Thus, repeated injection in the context of our work could differentially alter the emotional reactivity of our inbred mice, such that our repeatedly injected animals become low emotional responders. This could also account for why injection stress seems to override the effects of social isolation within the combined paradigm and why no apparent additive effect was observed for these animals.

Regarding the independent impact of permanent social isolation, it is unsurprising that this particular stressor, which is more ethological in nature, promotes aberrant behavioural and biological changes across multiple domains, especially in a gregarious species. Mice are social due to the various advantages that an organized social structure provides in terms of mating selection, resource allocation, and social status^109^. Therefore, by socially isolating animals that are biologically and evolutionarily suited to social living, it is understandable how the removal of social structure could impair physiology, and/or physiological responses that then ultimately manifest into aberrant behaviours. Indeed, preclinical research consistently shows how chronically isolated rodents have increased anxiety- and depressive-like behaviour^8,11,110^, together with altered HPA axis activity^4,11,110^, and specific increases in the pro- vs. anti-inflammatory balance, i.e., increased TNFα/IL6 and decreased IL2^2,111^—outcomes all observed in our socially isolated animals. Moreover, in humans, who are also a social species, the lack of social support is an important risk factor for affective disorders and social isolation/loneliness has been consistently associated with depression^112**–**115^. Interestingly, a recent study even demonstrates how social integration can be protective against inflammation in young black women^116^.

Although our work was designed to ensure maximum validity, it is unknown how generalisable our findings are relative to wider animal research when the species/strain, age and sex of the animal can impact both the behavioural and neurobiological phenotypes associated with stress exposure^117**–**120^. Due to financial and time constraints for such a huge programme of research, we only used young adult male animals for our research, which represents a limitation particularly when the prevalence of depression is significantly higher in women, in adolescence and old age^121**–**123^, and when research using female rodents is underrepresented^124,125^. However, given that males are less susceptible to clinical depression, one could speculate that in the context of our work, female BALB/c mice could be equally, if not more, sensitive to the impact of the different types of stress. Future work should prioritise investigating the impact of different stressors in both male and female animals and in different strains/species, and it is our ambition to extend our work to female animals in the near future.

Moreover, it remains unknown whether the observed biological changes are direct consequences of chronic stress exposure, indirect changes from other biological system alterations, or simply adaptive compensation. By characterizing biological phenotypes at the end of treatment, based on a single snapshot, we cannot determine the temporal sequence of these changes, and more importantly, may have missed earlier biological alterations. Future work should aim to collect behavioural readouts, blood samples, and tissue from parallel groups to determine the temporal sequence of these reported outcomes and more fully explore the mechanistic links.

Despite these limitations, this is the first time that a study has assessed all of these biological processes together and done an extensive evaluation of both microglial biology and neurogenesis. Moreover, we have also taken into account the importance of the hippocampal dorsal-ventral axis^126^.

In summary, the outcomes of our study provide some novel insight into the changes that occur in behaviour, in peripheral stress and inflammatory systems, and within the brain, following exposure to different types of chronic stress, and how the type of stress can make a difference in terms of these changes. We demonstrate that the type of stress exposure could differentially alter depressive symptomology and/or its biological basis, thus informing the molecular underpinning of the clinical studies showing that different types of abuse and maltreatment can differentially affect mental health. Moreover, our work gives clinically-relevant insights into the effects of social isolation, a condition that can increase morbidity and mortality^127,128^ and can specifically lead to MDD^112**–**115^, consistently with our behavioural data, while the relationship between repeated injections and anxiety may be relevant to the impact of recurrent medical treatments on mental health. Finally, given the global impact of the coronavirus outbreak, for which social isolation has increased significantly as a consequence, understanding the psychological and biological effects of social isolation has become fundamentally important in further understanding, and then subsequently alleviating, the impact that social isolation may have worldwide.

## Supplementary information

Supplementary Material
