# Supplementary material for: The type of stress matters: repeated injection and permanent social isolation stress in male mice have a differential effect on anxiety- and depressive-like behaviours, and associated biological alterations
Source: Transl Psychiatry. 2020 Sep 21;10:325. doi: 10.1038/s41398-020-01000-3 (PMC7505042; doi:10.1038/s41398-020-01000-3)
Supplement: Supplementary file 1 — Supplementary Material [file 41398_2020_1000_MOESM1_ESM.docx]

**SUPPLEMENTARY MATERIAL**

**MATERIALS AND METHODS**

**Intraperitoneal injection procedure**

Intraperitoneal (i.p.) injections were carried out according to recommended guidelines, and as previously described [1, 2]. Briefly, animals were scruffed and injected using a 1ml syringe with a 25-gauge needle. The injection process lasted approximately 1-2 seconds, with saline volumes of 10ml/kg body weight administered between 10:00 and 12:00 daily. A new needle was used for each animal, and the site of peritoneal administration, that is, on the left or right side, was alternated on a daily basis. Injection naïve animals were handled, but not injected.

**Physical health assessment**

Body weight and food intake was measured weekly during the first six weeks of stress exposure.

**Body weight**

Body weight was reported as absolute body weight and as a percentage weight change relative to baseline. Baseline measures were taken on arrival, and subsequent measurements were obtained on a weekly basis.

**Food intake**

Food intake was also measured and reported as an absolute change in the amount of food consumed over a 24h period on a weekly basis. For animals housed in sibling pairs, food intake was averaged out across the pair.

**Behavioural assessment**

**General conditions**

Behavioural testing was carried out by the same experimenter, under stringent environmental control, using standardised test procedures. No husbandry procedures were performed before, or on, the day of behavioural testing as cage changing can typically increase general activity and stress levels in rodents [3, 4]. Testing was typically conducted between 09:00 and 15:00, with each behavioural task being carried out over a 2-day period, so that a minimum 24h period of rest could be included for each animal between rounds of testing as per recommendations [5, 6]. The test order of experimental groups was completely randomized to control for diurnal variations, and animals were allowed to habituate to the experimental room for at least 1h prior to testing. The order in which each assay was carried out was based on previous recommendations [1, 5], starting with the tasks requiring maximal levels of novelty (anxiety tests) and ending with the most stressful tasks.

**Behavioural assays**

***Open Field Test (OFT)***

The OFT is specifically designed to assess anxiety-like behaviour in rodents [7, 8]. Mice were placed in a 40cm diameter round arena, evenly illuminated with white light (10 lux) for ten minutes. The arena was virtually divided into three zones: the centre zone (20cm diameter), inner zone (30cm diameter) and the remaining outer zone, using EthoVision software (EthoVision Program 3.1, Noldus, Wageningen, The Netherlands). EthoVision was used to determine the number of entries into, time spent in, and latency to enter, the centre zone of the arena. Avoidance of the centre zone represents an anxious phenotype. In addition, the total distance moved in the outer zone was also assessed as a measure of locomotor activity.

***Novelty Suppressed Feeding Test (NSFT)***

This behavioural assay evaluates both anhedonia and anxiety-like behaviour, as mice are placed into a situation where their desire to eat conflicts with avoidance of a novel, potentially threatening environment [9]. This assay was carried out as previously described [10]. Mice were food deprived, but not water deprived, 24h prior to testing, and then placed into a brightly lit (300 lux) open arena (45x32x20cm) for five minutes, with a food pellet securely attached to a white card in the centre of the arena. Latency to feed and food consumption in the arena was measured to assess both anhedonic and anxiety-like behaviour, with a longer latency to feed time representing a more anxious phenotype.

***Sucrose Preference Test (SPT)***

The SPT, which essentially measures a rodent’s appetite for a highly palatable, rewarding substance, has been extensively used to assess anhedonia in chronic stress models, with decreased intake and/or preference for the palatable solution a behavioural measure of hedonic deficit [11, 12]. The SPT was carried out as previously described [13]. One week prior to testing, experimental groups underwent three days of habituation with 2.5% sucrose solution in order to establish individual preference. After habituation mice were given (between 19:00 and 9:00) a free choice between two bottles for 14h, one with 1% sucrose solution and another with fresh tap water. The test was repeated 24h later for another 14h period, where the bottles were switched to balance the effect of side preference in drinking behaviour. Animals were not deprived of food or water before each test. Two water bottles were in place between sucrose testing. An average sucrose preference was assigned for pair-housed animals, as they were not separated during testing to control for the effects of periodic social isolation. Bottles were weighed each day to calculate the consumption of water, sucrose solution, and total liquid intake for all experimental animals. Food intake was also measured as a potential covariate. The preference for sucrose was calculated as a percentage of the consumed sucrose solution relative to the total amount of liquid drunk. After both test days, average two-day sucrose preference rates were calculated for each cage. To minimize the wastage of liquids during each sucrose test, we implemented recommendations as previously described [14]. Additionally, to account for any wastage during testing, ‘wastage tests’ were performed. Briefly, three wastage cages, each with a sucrose and water bottle, were set up for each rack. Wastage cages were positioned randomly in different areas of the rack and bottles were weighed before and after each SPT trial to estimate average liquid wastage.

***Porsolt Swim Test (PST)***

This behavioral assay was carried out according to the Porsolt’s procedure [15, 16] and is considered to be a reflection of behavioural despair in an inescapable stress situation, with increased immobility in the arena representing a depressive-like phenotype. Mice were introduced to a clear perspex cylinder (50x15x15cm) filled with 40cm of water at room temperature, with a dim white light illumination (10 lux), for six minutes on two consecutive days between 09:00 and 12:00. Behaviour was recorded and manually scored using Ethovision (Ethovision Program 3.1, Noldus, Wageningen, The Netherlands). Behavioural parameters, which included swimming (horizontal posture with limb movement), struggling (vertical posture with limb movement), and immobility (the absence of movement except that necessary to keep afloat) were all assessed with the latency, frequency and duration for each parameter recorded.

***Social Dominance Tube Test***

To examine the potential effects of social hierarchy on behavioural outcomes the dominance tube test was implemented for pair housed experimental groups. This behavioural assay was performed as previously described [17]. Sibling pairs were placed headfirst at opposite ends of a clear plastic tube (30x3.3x3.5cm), positioned in the centre of a dimly lit (5 lux) open arena (45x32x20cm), and simultaneously released. Dominant animals were recognized as those that forced the subordinate completely out of the tube within two minutes. The test was repeated three times, over four weeks, before assigning hierarchal status to animals.

***The buried food test***

This assay was carried out as previously described [18]. It relies on the animal's natural tendency to use olfactory cues for foraging, the main parameter of which is the latency to uncover a small piece of cookie hidden beneath a layer of cage bedding, within a limited time period. For four days, prior to testing, experimental groups were habituated to cookie pieces (two daily; Cookie Crisp Cereal, Nestle, Switzerland) in order to overcome any potential food neophobia. After habituation, mice were placed into clean tecniplast cages (32x16x14cm) with a 3cm layer of fresh sawdust, dimly lit (5 lux), with a cookie buried to the depth of 1cm at the top end of the arena either on the left, right or in the centre. Latency to find the cookie was measured, with the time capped at 15 minutes. Mice were not food deprived and were allowed to acclimatise to each test arena for five minutes prior to testing.

**Biochemical assessment**

**Blood sampling**

Blood was sampled at two time points in the final week of behavioural testing for all animals. Sampling took place between 08:30 and 12:30, and more specifically 24h before, and 30 minutes after, the PST. To control for diurnal variation the sampling order was randomised across experimental groups, and to minimise the confounding effects of the procedure itself on corticosterone measures, all blood samples were collected within three minutes, before activation of the HPA axis [19]. Before sampling, animals were habituated just outside the procedure room for 30 minutes, after which whole blood was subsequently collected by incision method from the lateral tail vein as previously described [20].

**Corticosterone measurement**

Plasma corticosterone levels were measured in duplicates using commercially available corticosterone enzyme-linked immunosorbant assay kit (Enzo Life Sciences, Lausen, Switzerland) according to the manufacturer’s instructions for small sample volume. Briefly, 10μl of each plasma sample was diluted 1:40 with steroid displacement reagent and ELISA buffer. Next, five corticosterone standards were made from a 200ng/ml corticosterone standard solution using a serial dilution method, thereby generating five corticosterone standards of 20, 4, 8, 1.6 and 3.2ng/ml. Blank samples, standards and diluted biological samples were then transferred directly onto the ELISA microtitre plate pre-coated with corticosterone antibody (donkey anti-sheep IgG). Corticosterone conjugate, a solution of alkaline phosphatase conjugated with corticosterone, and a solution of sheep polyclonal antibody to corticosterone, were subsequently added, and the plate incubated for two hours at room temperature on a plate shaker moving at 220rpm. After incubation, the plate contents were discarded, and the plate washed three times with tris-buffered saline. Finally, a substrate solution of p-nitrophenyl phosphate was added, and the plate was incubated at room temperature without shaking for one hour, before being terminated with a stop solution of trisodium phosphate. Absorbance was immediately read at a wavelength of 405nm using a DTX 880 Multimode Detector (Beckman-Coulter). Data were analysed using a four parameter logistic algorithm to derive concentrations of samples from known standards using the “my assays” online data tool (MyAssays Ltd., <http://www.myassays.com/five-parameter-logistic-curve.assay>). Inter-assay variability was estimated at %CV=4.6, while the average intra-assay variability for duplicates was %CV=7.4. Biological samples with a CV above 15% were repeated, or otherwise excluded from further analyses as per recommendations [21]. ELISA kit sensitivity was reported to be 27.0 pg/ml (range 32 - 20,000 pg/ml).

**Cytokine assessment**

Custom-made mouse premixed multi-analyte kits were used (R&D systems, Minneapolis, USA), according to the manufacturer’s instructions. Briefly, 20μl of each plasma sample was diluted 1:5 with calibrator diluent, which was a buffered protein base with preservatives. Next, six standards were made by a 1:3 serial dilution, generating standard curves for each of the ten analytes. Standards and samples were then incubated with microparticle cocktail solution, which contained ten antibodies to the ten chosen analytes, for two hours, in the dark, at room temperature, and on a plate shaker operating at 800rpm. All efforts were made to minimise light quenching of the fluorochrome by covering plates with foil throughout the procedure. After incubation, the plate was washed three times with wash buffer, specifically; buffered surfactant, and samples were subsequently incubated with diluted biotinylated detector antibody for one hour as per the first incubation stage. After the second incubation step, the plate was washed as previously described, and then incubated with diluted streptavidin-PE solution, which was streptavidin conjugated to the fluorescent protein, phycoerythrin, for 30 minutes under exactly the same previously described incubation conditions. Finally, the plate was washed, microparticles were resuspended with wash buffer, and the plate was read using the Luminex 200 platform. Data were analysed using a five parameter logistic algorithm to derive concentrations of samples from known standards using the “my assays” online data tool (MyAssays Ltd., <http://www.myassays.com/five-parameter-logistic-curve.assay>). Inter-assay variability was estimated at %CV=5.5, while the average intra-assay variability for duplicates was %CV=4.5. The lower detection limit for each analytes was as follows: IL-1r-assay vpg/ml; IL-2 = 0.48 pg/ml; IL-4 = 53.0 pg/ml; IL-6 = 2.3 pg/ml; IL-10 = 8.2 pg/ml; IL-12 = 12.8 pg/ml; CRP = 13.3 pg/ml; VEGF = 3.96 pg/ml; IGF = 3.46 pg/ml; TNF-α = 1.47 pg/ml.

**Histological assessment**

**Tissue collection**

Under terminal anaesthesia, animals were transcardially perfused and brains quickly extracted as previously described [22]. Briefly, a rapid, controlled and uniform fixation procedure using 4% paraformaldehyde (PFA, Sigma-Aldrich, Poole, UK) in 0.1M phosphate-buffered saline (PBS, pH 7.4, from PBS tablets, Gibco by Life Technologies, Paisley, UK) was performed for each animal and intact brains were carefully removed. Brains were post-fixed overnight in 4% PFA at 4°C, and subsequently stored in 30% sucrose solution in PBS at 4°C.

**Brain tissue sectioning**

All brain tissue was coronally sectioned at a thickness of 40μm using a Leitz freezing microtome (Microm HM430, Carl Zeiss Ltd, Cambridge, UK) as previously described [23, 24]. Coronal sections were stored in six-well plates in an anti-freeze cryoprotectant solution (30% glycerol, 15% sucrose and 0.05% sodium azide in tris-buffered saline [TBS]), and kept at 4°C, until required for immunoperoxidase or immunofluorescent labelling.

**Free-floating immunoperoxidase labelling**

For immunohistochemistry on free floating sections every sixth section was used. Briefly, sections were washed in TBS, incubated in 1% hydrogen peroxide (H_2_O_2_) solution in methanol for 30 minutes at room temperature (RT), washed, incubated for 1h at RT in 10% normal goat serum (NGS) in TBS with 0.25% TritonX (TBST), and then incubated overnight at 4°C in 10% NGS in TBST with primary antibody (rabbit anti-Ki67 1:200, ab15550, Abcam, USA; rabbit anti-DCX 1:1000, ab18723, Abcam, USA; rabbit anti-Iba1, 1:2500, 019-19741, Wako, Japan; rabbit anti-GFAP 1:3000, Z0334, Dako, Denmark; rabbit anti-s100β, 1:1000, ab52642, Abcam, USA). Following primary antibody incubation, sections were then washed, incubated for 2h at RT in 10% NGS in TBST with secondary antibody (goat anti-rabbit, 1:1000; Vector Laboratories, UK), washed, and then incubated in avidin-biotin complex for 2h at RT. Finally, sections were washed, and staining was subsequently visualised by incubating sections in 0.05% 3,3-diaminobenzidine tetrahydrochloride (DAB) and 0.001% H_2_O_2_ in TBS for 1-3 minutes, depending on the target antigen. All wash steps throughout the protocol included a triple incubation in 0.1M TBS (pH 7.6) for five minutes. Stained tissue sections were then mounted in anatomical order, counterstained with Harris’ haematoxylin for easier anatomical identification (Iba1 and GFAP stained tissue only), dehydrated in serial ethanol dilutions, defatted in xylene, and lastly coverslipped with distyrene plasticiser xylene mountant. For representative images of the various immunohistochemical stains see **Supplementary Figure 1**.

**S. Figure 1. Representative images of immunohistochemistry stains in the dentate gyrus of the hippocampus**

**A.**

**B.**

**C.**

**D.**

**E.**

**Ki67**

**DCX**

**IBA1**

**GFAP**

**S100β**

**40μm**

**40μm**

**40μm**

**40μm**

**40μm**

**40μm**

**20x**

**20x**

**20x**

**20x**

**20x**

**20x**

**20x**

**20x**

**20x**

**20x**

**40μm**

**40μm**

**40μm**

**40μm**

**(A). Top:** Ki67 negative control counterstained with haematoxylin. 20x magnification. Scale bar = 50μm. **Bottom:** Ki67 positive stain from representative control animal. 20x magnification. Scale bar = 40μm. **(B). Top:** DCX negative control counterstained with haematoxylin. 20x magnification. Scale bar = 50μm. **Bottom:** DCX positive stain from representative control animal. 20x magnification. Scale bar = 40μm. **(C). Top:** Iba1 negative control counterstained with haematoxylin. 20x magnification. Scale bar = 50μm. **Bottom:** Iba1 positive stain counterstained with haematoxylin taken from representative control animal. 20x magnification. Scale bar = 40μm. **(D). Top:** GFAP negative control counterstained with haematoxylin. 20x magnification. Scale bar = 50μm. **Bottom:** GFAP positive stain counterstained with haematoxylin taken from representative control animal. 20x magnification. Scale bar = 40μm. **(E). Top:** S100β negative control counterstained with haematoxylin. 20x magnification. Scale bar = 50μm. **Bottom:** S100β positive stain from representative control animal. 20x magnification. Scale bar = 40μm.

**Abbreviations:** DCX, Doublecortin; Iba1, Ionizing calcium-binding adapter molecule 1; GFAP, Glial fibrillary acidic protein.

**Stereological analyses of volume and immunopositive cell density**

Briefly, an Axioskop 2 MOT Zeiss microscope and a semiautomatic stereology system were used to estimate the volume of each region of interest (ROI), i.e., the dentate gyrus (DG) and prefrontal cortex (PFC), and the total number of immunopositive cells in that ROI. The ROI for each section was delineated under 10x magnification using defined anatomical landmarks and overlaid with a grid (X=94.6μm; Y=182.5μm) from which an unbiased selection of virtual counting frames was extracted. The size of each counting frame was set at 50x50μm, and there were 50 desired sampling sites per ROI based on parameters previously validated [25]. Using this systematic random sampling method, immunopositive cells within each sampling site were then identified at 40x magnification, and the optical fractionator probe with a dissector height of 15μm was used to manually count the cells in each ROI per the acceptance and rejection guidelines of the counting frame [26]. For each section, ROI volume was then subsequently estimated according to the Cavalieri principle [27], and unbiased cell population estimates were obtained by extrapolating the number of cells counted within the counting frames to the total estimated volume of each ROI [28]. Finally, immunopositive cell density was subsequently calculated by dividing the estimated cell population number by the estimated volume of the ROI. The sampling approach used, that is, the number of sections analysed per mouse, varied depending on the antigen, and was determined by the coefficient of error (CE), which is a measure of the precision of the cell population estimate, as influenced by the number and dispersion of the antigen across sampled sections [29]. In all instances, the CE was capped at 20%, and as such the number of sections sampled ranged from 4-11 sections/mouse (i.e., 11-Ki67, average CE=20%; 8-DCX, average CE=10%; 4-Iba1, average CE=8%; 6-GFAP average CE=10%, 6-s100β, average CE=10%). For all stereological analyses the experimenter was blinded to stress exposure group status.

**Threshold analyses of immunoreactivity for Iba1 and GFAP positive cells**

Blinded for exposure/group status, 30 unbiased non-overlapping images, across six sections/mouse were captured at 40x magnification from the DG and PFC using a macro (Lim, KCL; [https://github.com/yaumun/imagej-particle-analysis](https://eur03.safelinks.protection.outlook.com/?url=https%3A%2F%2Fgithub.com%2Fyaumun%2Fimagej-particle-analysis&data=01%7C01%7Candrea.du_preez%40kcl.ac.uk%7C4a445b94f5cd4c54e60d08d7352621df%7C8370cf1416f34c16b83c724071654356%7C0&sdata=xgIlg%2FBEueaV4FIf4H%2BoeDNWaF7M5hVoRZs4jsNArdg%3D&reserved=0)) and ImageJ software v1.51 [30]. Specifically, from each delineated ROI, images of five random sampling frames, each 200x200μm in size, were captured based on random coordinates generated from a python script (Lim, KCL). Using thresholding analysis in ImageJ, an appropriate threshold was then chosen to distinguish between positive immunostaining and non-specific background and was typically set at 0 for all images/mouse. All images were processed according to the same basic parameters (hue: 0-30; brightness: mean thresholding method), and a percentage surface area immunoreactivity score was subsequently generated for each captured image.

**Morphometric analyses of Iba1 positive cells**

Blinded for exposure/group status, images of Iba1 labelled cells were imported into ImageJ and single, whole Iba1 cells were isolated and traced at 40x magnification. Sholl analysis was performed for each cell image using the ShollAnalysis plugin (v.3.6.2; <http://imagejdocu.tudor.lu/>), using the log-log Sholl method as per recommendations [31], with the starting radius, step size and ending radius set as 10μm, 5μm, and 150μm, respectively, and selecting a linear profile. Cell images were also converted into binary images and skeletonized, and the AnalyzeSkeleton plugin (<http://imagejdocu.tudor.lu/>) was then used to collect data on average branch length and branch number. Cell soma area measurements, and the overall volume occupied by each Iba1 cell were also obtained using the ImageJ “measure” tool.

**Immunofluorescent double labelling of GFAP and SOX2**

Immunofluorescent double labelling was performed on 1 in 6 slide-mounted coronal sections. Mounted sections were first rehydrated in graded dilutions of ethanol, and then washed in distilled water before undergoing heat induced epitope retrieval, using sodium citrate buffer. Sections were then washed in distilled water, triple washed in TBS, incubated for 1h at RT in 10% normal donkey serum (NDS) in TBST, and then incubated overnight at 4°C in 10% NDS in TBST with both primary antibodies (rabbit anti-SOX2, 1:400, ab5603, EMD Millipore, Merck, USA; goat anti-GFAP, 1:500, ab53554, Abcam, USA). The following day, sections were triple washed, incubated with both secondary antibodies (donkey anti- rabbit, Alexa Fluor 488, 1:200; A-21206, donkey anti-goat, Alexa Fluor 594, 1:200 A-11058; both Life Tech, USA) for 2h at RT, triple washed again, and incubated in 0.3% Sudan Black B solution in ethanol. Sections were subsequently tripled washed, and finally incubated with 1 μg/mL Hoescht 33342 solution in water for ten minutes, in the dark, before being washed for the final time with distilled water. TBS (pH 7.6) was used for all wash steps, unless otherwise specified, at five-minute intervals. Immediately after staining, slides were coverslipped with 50μl anti-fading mowiol mountant, and left to cure in the dark, overnight, at RT. For representative images of the immunofluorescent stain see **Supplementary Figure 2**.

**Immature neuron classification based on DCX morphology**

DCX positive cells were classified according to four neuroblast subtypes based on their level of maturation (**Supplementary Figure 3**) and previous research [32]. Specifically, DCX positive cells in their most immature form, that is, the proliferative neuroblasts, were defined as DCX positive cells with no, or short, plump processes. The next subtype and subsequent stage of maturation is signified by the intermediate neuroblasts, which were classified as DCX positive cells with medium length processes without branching.

Early stage postmitotic neuroblasts were defined as DCX positive cells with one strong dendrite branching out into the molecular layer, and finally the most mature form of neuroblasts, which are classified as the late stage postmitotic neuroblasts, were defined as cells with delicate dendritic tree branching extending to the granule cell layer. The cell density for each neuroblast type in both the dorsal and ventral DG was determined using stereological analysis as described.

**SOX2**

**Hoescht**

**A.**

**B.**

**C.**

**GFAP**

**COMPOSITE**

**D.**

**40x**

**40x**

**40x**

**20x**

**S. Figure 2. Representative images of immunofluorescent stains in the dentate gyrus of the hippocampus**

**(A)** All cell nuclei stained with Hoescht (blue); **(B)** All progenitor cells stained for SOX2 (green); **(C)** All glial fibrillary acidic protein (GFAP) positive cells (red). All images were taken from a representative control animal at 20x magnification (D) and 40x magnification (A-C). Scale bar = 20μm.

**S. Figure 3. Classification guide of neuroblast cell morphology**

Plumpe and colleagues (2006)^42^ classification guide to determine what immature neuron morphology types were present in the dorsal and ventral dentate gyrus of the hippocampus was used and adapted such that immature neurons were classified according to the four morphology stages as depicted in the schematic.

**Rationale for the use of young adult, male BALB/C mice**

Male BALB/c mice were selected due to their sensitivity to stress [33] and their susceptibility to the development of anxiety and depressive-like behaviour [34, 35], making them an ideal mouse strain for detecting exposures that will ameliorate these behavioural phenotypes. Unfortunately, female mice were not additionally assessed due to time and logistical constraints, as using females would have required groups housed in female only housing rooms, which were not available. Given the size and scope of the work carried out, this would not have been practical or feasible. Finally, although depression can develop at any age between early childhood and older adulthood, both national and cross-national epidemiological studies report that the first onset of depression most frequently occurs in the 20s to early 30s [36–38]. Therefore, the animal equivalent of a young adult was purposely selected for study.

**Statistical analyses**

*Data analysis protocols*

Body weight data were analysed using a repeated measures two-way ANOVA with ‘stress group’ as the between subjects variable (four levels) and ‘time’ as the within subjects variable (six levels).

Behavioural readouts were analysed using the one-way ANOVA where appropriate for group comparisons with ‘stress group’ as the between subjects variable (four levels).

All plasma readouts were analysed using a repeated measures two-way ANOVA with ‘stress group’ as the between subjects variable (four levels) and ‘time’ as the within subjects variable (two levels: pre and post-FST).

Immunoreactivity, cell density and/or immunofluorescent data in the hippocampus were analysed using independent *t* tests or two-way ANOVA with ‘stress group’ as the between subjects variable (four levels) and ‘region’ as the within subjects variable (two levels: dorsal and ventral). Immature neuron and GFAP classification, and associated cell density, data were analysed using three-way factorial ANOVA with ‘stress group’ as the between subjects variable (two or four levels) and ‘region’ and ‘DCX/GFAP classification’ as the within subjects variables (region: two levels, dorsal and ventral; DCX classification: four levels; GFAP classification: two levels). Data for all immunohistochemical parameters pertaining to the PFC were analysed using one-way ANOVA, or the independent samples *t*-test for group comparisons. Iba1 morphometric analyses were analysed using a repeated measure general linear model (GLM) to control for any clustering effects associated with sampling, with ‘stress group’ as the between subjects variable (two levels: control and stress), and ‘Iba1 cell’ as the repeated measure.

**RESULTS**

**LOCOMOTION**

**A**

**B**

**OLFACTION**

**C**

**LIQUID AND FOOD CONSUMPTION**

**D**

**E**

**S. Figure 4. Effect of locomotion, olfaction, and food and liquid consumption on behavioural performance (A)** mean (± SEM) locomotor activity in the OFT (F [3,46] = 1.4; *p* = .25), **(B)** median (± IQR) locomotor activity in the NSFT (K [4,50] = 6.0; *p* = .11), **(C)** median (± IQR) olfaction in the Buried Food/Cookie Test (K [4,50] = 2.4; *p* = .31), **(D)** mean (± SEM) total liquid consumption in the SPT (F [3,46] = 2.2; *p* = .11), **(E)** mean (± SEM) food consumption in the SPT (F [3,46] = 1.8; *p* = .16). No significant differences were observed for any of the measures between experimental groups. Analyses: One-way ANOVA or Kruskal Wallis with Bonferroni or Dunn’s *post hoc* correction, respectively.

| **S.Table 1**. Effect of social hierarchy on altered behavioural outcomes in the OFT, NSFT and PST | | | | | | |
| --- | --- | --- | --- | --- | --- | --- |
| **Variable** | **Control animals (n=20)** | | | **Physically stressed animals (n=10)** | | |
|  | Dominant Mean/Mean rank (SE) | Subordinate  Mean/Mean rank (SE) | *p* value | Dominant Mean/Mean rank (SE) | Subordinate  Mean/Mean rank (SE) | *p* value |
| **OFT** |  |  |  |  |  |  |
| Centre duration | MR: 5.0 | MR 6.0 | 0.69 | MR: 6.2 | MR: 4.8 | 0.55 |
| **NSFT** |  |  |  |  |  |  |
| Latency to feed | MR: 6.3 | MR: 4.7 | 0.42 | MR: 5.8 | MR: 5.2 | 0.84 |
| **PST** |  |  |  |  |  |  |
| Immobility duration | 148.2 (13.6) | 122.9 (7.8) | 0.15 | 178.6 (21.4) | 160.6 (18.5) | 0.54 |
| Note: Effect of hierarchy in the sucrose preference test could not be measured as an average measurement for the pair was taken  Analyses: Independent samples *t* test or Mann Whitney test | | | | | | |

**B**

**A**

**OPEN FIELD TEST**

**NOVELTY SUPRESSED FEEDING TEST**

**D**

**C**

**PORSOLT SWIM TEST**

**E**

**F**

**G**

**H**

**I**

**J**

**S. Figure 5. Effect of repeated injection, social isolation, and combined stress on various behavioural parameters in the Open Field Test, Novelty Suppressed Feeding Test, and Porsolt Swim Test (A)** median (± IQR) latency to enter the centre in the OFT (K [4, 50] = 3.8, *p* = .29), **(B)** mean (± SEM) frequency to enter the centre in the OFT (F [3,46] = 7.7, *p* < .001), **(C)** mean (± SEM) arena food consumption in the NSFT (F [3,46] = 4.7, *p* = .006), **(D)** mean (± SEM) homecage food consumption in the NSFT (F [3,46] = 1.1; *p* = .77), **(E)** median (± IQR) latency to become immobile in the PST (K [4,50] = 6.0; *p* = 0.11), **(F)** mean (± SEM) immobility frequency in the PST (F [3,46] = 4.6; *p* = .007), **(G)** mean (± SEM) swimming duration in the PST (F [3,46] = 9.5, *p* < .001), **(H)** mean (± SEM) struggling duration in the PST (F [3,46] = 2.3; *p* = .09), **(I)** median (± IQR) latency to struggle in the PST (K [4,50] = 4.9; *p* = .18), **(J)** mean (± SEM) struggling frequency in the PST (F [3,46] = 2.4; *p* = .08). * *p* < .05; ** *p* < .01; *** *p* < .001 relative to control (adjusted *p* values). Analyses: One-way ANOVA or Kruskal Wallis with Bonferroni or Dunn’s *post hoc* correction, respectively.

| **S.Table 2.** Summary of all the behavioural changes associated with chronic stress exposure | | | |
| --- | --- | --- | --- |
| **Variable** | **Physical stress** | **Psychosocial stress** | **Combined stress** |
| **Anxiety-like behavioural measures** |  |  |  |
| OFT: Centre duration  (See **Figure 2.A**) | **-** | **-** | **-** |
| OFT: Centre latency  (See **S. Figure 6.A**) | = | = | = |
| OFT: Centre frequency  (See **S. Figure 6.B**) | **-** | **-** | **-** |
| OFT: Locomotor activity  (See **S. Figure 5.A**) | = | = | = |
| NSFT: Latency to feed  (See **Figure 2.B**) | **+** | **+** | **+** |
| NSFT: Arena food consumption  (See **S. Figure 6.C**) | **-** | **-** | = |
| NSFT: Homecage food consumption (See **S. Figure 6.D**) | = | = | = |
| NSFT: Locomotor activity  (See **S. Figure 5.B**) | = | = | = |
| NSFT: Olfactory ability  (See **S. Figure 5.C**) | = | = | = |
| **Anhedonic-like behavioural measures** |  |  |  |
| SPT: Sucrose consumption  (See **Figure 2.C**) | = | **-** | = |
| SPT: Total liquid consumption  (See **S. Figure 5.D**) | = | = | = |
| SPT: Food consumption  (See **S. Figure 5.E**) | = | = | = |
| **Depressive-like behavioural measures** |  |  |  |
| PST: Immobility duration  (See **Figure 2.D**) | = | **+** | = |
| PST: Immobility latency  (See **S. Figure 6.E**) | = | = | = |
| PST: Immobility frequency  (See **S. Figure 6.F**) | = | = | = |
| PST: Swimming duration  (See **S. Figure 6.G**) | = | **-** | = |
| PST: Struggling duration  (See **S. Figure 6.H**) | = | = | = |
| PST: Struggling latency  (See **S. Figure 6.I**) | = | = | = |
| PST: Struggling frequency  (See **S. Figure 6.J**) | = | = | = |
| Key:  + (shaded green); Represents a significant increase in readout relative to control  - (shaded blue); Represents a significant decrease in readout relative to control  = (unshaded); Represents no significant change in readout relative to control | | | |

**A**

**B**

**S. Figure 6. Effect of repeated injection, social isolation, and combined stress on inflammatory profiles of interleukin (IL) 6 and IL12 (A)** mean (± SEM) plasma IL6 levels before and after acute stress exposure, **(B)** mean (± SEM) plasma IL12 levels before and after acute stress exposure. No significant differences were observed in plasma levels of IL6 or IL12 between (or within) experimental groups. Analyses: Repeated measures two-way ANOVA with Bonferroni *post hoc* correction.

| **S.Table 3.** Detailed summary of all the corticosterone and systemic inflammatory changes associated with chronic stress exposure | | | | | |
| --- | --- | --- | --- | --- | --- |
| **Variable** | **Physical stress** | **Psychosocial stress** | **Combined stress** | **Statistics:**  **Repeated measures two-way ANOVA** | **Figure reference** |
| **Stress responsivity** |  |  |  |  |  |
| Corticosterone pre-PST | = | = | = | **Effect of chronic stress exposure:**  F [3,92] = 10.6, *p* < .001;  **Effect of acute stress:**  F [1,92] = 317.5, *p* < .001;  **Interaction:**  F [3,92] = 5.1, *p* = .003 | Figure 3.A |
| Corticosterone post-PST | **41% > all groups** | **22% < all groups** | = |  |  |
| Corticosterone pre- *vs*. post-PST | **258%+** | **218%+** | **275%+** |  |  |
| **Systemic inflammation** |  |  |  |  |  |
| TNFα pre-PST | **35% < controls** | **39% > all groups** | = | **Effect of chronic stress exposure**:  F [3,92] = 21.8, *p* < .001;  **Effect of acute stress**:  F [1,92] = 3.6, *p* = .06  **Interaction:**  F [3,92] = 3.2, *p* = .03 | Figure 3.B |
| TNFα post-PST | = | **28% > physical stress**  **35% > controls** | = |  |  |
| TNFα pre- *vs*. post-PST | = | = | = |  |  |
|  |  |  |  |  |  |
| IL1β pre-PST | = | **4.7% < controls**  **3.8% < combined stress** | = | **Effect of chronic stress exposure:**  F [3,92] = 9.7, *p* < .001  **Effect of acute stress:**  F [1,92] = 2.9, *p* = .09  **Interaction:**  F [3,92] = 4.1, *p* = .01 | Figure 3.C |
| IL1β post-PST | = | = | **3% < controls** |  |  |
| IL1β pre- *vs*. post-PST | = | = | = |  |  |
|  |  |  |  |  |  |
| IL2 pre-PST | = | = | = | **Effect of chronic stress exposure:**  F [3,92] = 14.6, *p* < .001  **Effect of acute stress:**  F [1,92] = 12.6, *p* < .001  **Interaction:**  F [3,92] = 3.1, *p* = .03 | Figure 3.D |
| IL2 post-PST | = | **15% < controls**  **23% < combined stress** | = |  |  |
| IL2 pre- *vs*. post-PST | = | **15% +** | = |  |  |
|  |  |  |  |  |  |
| IL4 pre-PST | = | = | **19% < controls** | **Effect of chronic stress exposure:**  F [3,92] = 12.6, *p* < .001  **Effect of acute stress:**  F [1,92] < 0.01, *p* = .98  **Interaction:**  F [3,92] = 1.6, *p* = .20 | Figure 3.E |
| IL4 post-PST | **18% < controls** | **22% < controls** | **28% < controls** |  |  |
| IL4 pre- *vs*. post-PST | = | = | = |  |  |
|  |  |  |  |  |  |
| IL6 pre-PST | = | = | = | **Effect of chronic stress exposure:**  F [3,92] = 0.06, *p* = .99  **Effect of acute stress:**  F [1,92] = 0.01, *p* = .94  **Interaction:**  F [3,92] = 0.04, *p* = .99 | Supplementary Figure 7.A |
| IL6 post-PST | = | = | = |  |  |
| IL6 pre- *vs*. post-PST | = | = | = |  |  |
|  |  |  |  |  |  |
| IL10 pre-PST | = | **18% < all groups** | = | **Effect of chronic stress exposure:**  F [3,92] = 9.3, *p* < .001  **Effect of acute stress:**  F [1,92] = 0.02, *p* = .88  **Interaction:**  F [3,92] = 0.1, *p* = .98 | Figure 3.F |
| IL10 post-PST | = | **18% < all groups** | = |  |  |
| IL10 pre- *vs*. post-PST | = | = | = |  |  |
|  |  |  |  |  |  |
| IL12 pre-PST | = | = | = | **Effect of chronic stress exposure:**  F [3,92] = 0.9, *p* = .45  **Effect of acute stress:**  F [1,92] = 0.1, *p* = .74  **Interaction:**  F [3,92] = 1.1, *p* = .37 | Supplementary Figure 7.B |
| IL12 post-PST | = | = | = |  |  |
| IL12 pre- *vs*. post-PST | = | = | = |  |  |
| **Growth factors** |  |  |  |  |  |
| VEGF pre-PST | = | **13% < controls** | **13% < controls** | **Effect of chronic stress exposure:**  F [3,92] = 8.9, *p* < .001  **Effect of acute stress:**  F [1,92] = 21.5, *p* < .001  **Interaction:**  F [3,92] = 2.4, *p* = .08 | Figure 3.G |
| VEGF post-PST | = | = | **10% < controls** |  |  |
| VEGF pre- *vs*. post-PST | = | **15% +** | = |  |  |
| Key:  + (shaded green); Represents an increase in plasma levels; = (unshaded); Represents no change in plasma levels; < (shaded blue); Represents a decrease in plasma levels relative to; > (shaded green); Represents an increase in plasma levels relative to. Analyses: Repeated measures two-way ANOVA with Bonferroni *post hoc* correction. | | | | | |

| **S. Table 4.** Effect of social hierarchy on altered biochemical outcomes | | | | | | |
| --- | --- | --- | --- | --- | --- | --- |
| **Variable** | **Control animals (n=20)** | | | **Physically stressed animals (n=10)** | | |
|  | Dominant Mean (SE) | Subordinate  Mean (SE) | *p* value | Dominant Mean (SE) | Subordinate  Mean (SE) | *p* value |
| **Peripheral inflammation** |  |  |  |  |  |  |
| TNFα baseline | 0.042 (0.005) | 0.035 (0.002) | 0.25 | 0.031 (0.004) | 0.032 (0.005) | 0.96 |
| TNFα post stress | 0.031 (0.002) | 0.041 (0.008) | 0.22 | 0.028 (0.002) | 0.028 (0.001) | 0.75 |
| IL1β baseline | 1.41 (0.03) | 1.41 (0.04) | > 0.99 | N/A | N/A | N/A |
| IL1β post stress | 1.43 (0.04) | 1.40 (0.03) | 0.51 | N/A | N/A | N/A |
| IL2 post stress | 0.035 (0.004) | 0.035 (0.005) | 0.97 | N/A | N/A | N/A |
| IL4 baseline | 0.30 (0.002) | 0.30 (0.1) | 0.57 | N/A | N/A | N/A |
| IL4 post stress | 0.35 (0.003) | 0.30 (0.08) | 0.54 | N/A | N/A | N/A |
| IL10 baseline | 0.18 (0.06) | 0.18 (0.002) | 0.92 | 0.18 (0.02) | 0.18 (0.03) | 0.35 |
| IL10 post stress | 0.19 (0.1) | 0.18 (0.002) | 0.69 | 0.18 (0.01) | 0.18 (0.02) | 0.93 |
| **Growth factors** |  |  |  |  |  |  |
| Plasma VEGF baseline | 0.035 (0.003) | 0.034 (0.003) | 0.73 | N/A | N/A | N/A |
| Plasma VEGF post stress | 0.036 (0.002) | 0.037 (0.003) | 0.81 | N/A | N/A | N/A |
| **Hormones** |  |  |  |  |  |  |
| Corticosterone post stress | 94.7 (16.1) | 69.8 (18.9) | 0.37 | 13.3 (7.1) | 16.2 (17.1) | 0.16 |
| Analyses: Independent samples *t* test or Mann Whitney test | | | | | | |

**D**

**B**

**C**

**D**

**A**

**S. Figure 7. Effect of repeated injection, social isolation, and combined stress on neuroinflammatory profiles in the prefrontal cortex (A)** mean (± SEM) Iba1 positive immunoreactivity in the PFC (F [3,46] = 1.2; *p* = 0.33), **(B)** Representative photomicrographs of the PFC taken from control animals stained for Iba1 (whole coronal sections at 2x magnification; close up images at 20x magnification; scale bar = 50μm), **(C)** mean (± SEM) GFAP positive immunoreactivity in the PFC (F [3,46] = 1.0; *p* = 0.39). **(D)** Representative photomicrographs of the PFC taken from control animals stained for GFAP (whole coronal sections at 2x magnification; close up images at 20x magnification; scale bar = 50μm), No significant differences were observed in either Iba1 or GFAP immunoreactivity in the PFC between experimental groups. Analyses: One-way ANOVA with Bonferroni *post hoc* correction.

| **S. Table 5.** Morphological characteristics of Iba1 cells in the dentate gyrus of the hippocampus | | | | | | | |
| --- | --- | --- | --- | --- | --- | --- | --- |
| **IBA1 SHOLL, SKELETON AND SOMA ANALYSES** | | | | | | | |
|  | **Ventral Dentate Gyrus** | | | **Dorsal Dentate Gyrus** | | | |
|  | **Control** | **Repeated injection** | **Statistics** | **Control** | **Social isolation** | | **Statistics** |
| **Sholl Analysis Summary:** |  |  |  |  |  |  | |
| **Number of primary processes *(Np)*** | **4.5**  **(0.16) ***** | **5.42**  **(0.15) ***** | **F [1,73] = 20.1;**  ***p* < .001** | **7.7**  **(0.26) ***** | **5.96**  **(0.12) ***** | **F [1,70] = 36.3;**  ***p* < .001** | |
| **Intersections maximum *(Nm)*** | **14.66**  **(0.48) *** | **12.2**  **(0.59) *** | **F [1,68] = 7.4;**  ***p* = .02** | 15.28  (0.53) | 14.6  (0.45) | F [1,70] = 1.6;  *p* = .46 | |
| **Schoenen ramification index score *(Nm/Np)*** | **3.25**  **(0.1) ***** | **2.25**  **(0.1) ***** | **F [1,70] = 48.1;**  ***p* < .001** | **1.98**  **(0.11) ***** | **2.45**  **(0.05) ***** | **F [1,74] = 25.3;**  ***p* < .001** | |
| **Critical value** | **13**  **(3.16) *** | **11.53**  **(3.67) *** | **F [1,70] = 6.6;**  ***p* = .03** | 13.57  (0.48) | 12.77  (0.44) | F [1,73] = 1.9;  *p* = .32 | |
| **Critical radius** | **70.19**  **(2.19) *** | **62.20**  **(2.44) *** | **F [1,68] = 8.2;**  ***p* = .02** | 68.3  (2.54) | 68.89  (2.76) | F [1,70] = 2.1;  *p* = .12 | |
| **Mean value** | **7.26**  **(0.33) *** | **8.11**  **(0.25) *** | **F [1,74] = 6.8;**  ***p* = .03** | 8.68  (0.31) | 8.17  (0.26) | F [1,72] = 1.2;  *p* = .65 | |
| Regression (log-log) coefficient | 0.87  (0.03) | 0.87  (0.01) | F [1,70] < 0.1;  *p* = .99 | 0.89  (0.004) | 0.89  (0.004) | F [1,68] < 0.1;  *p* = .99 | |
| **Skeleton Analysis Summary:** |  |  |  |  |  |  | |
| **Average number of processes** | **82.94**  **(3.66) ***** | **61.83**  **(3.73) ***** | **F [1,70] = 16.4;**  ***p* < .001** | 64  (5.17) | 73  (2.31) | F [1,54] = 0.1;  *p* = .99 | |
| Average process length (μm) | 23.33  (0.6) | 23.91  (0.62) | F [1,51] = 0.4;  *p* = .51 | 26.07  (1.03) | 24.49  (0.46) | F [1,78] = 0.2;  *p* = .65 | |
| **Max process length (μm)** | **109.34**  **(3.75) **** | **90.04**  **(3.86) **** | **F [1,78] = 12.9;**  ***p* = .001** | 110.02  (5.59) | 104.29  (2.5) | F [1,70] = 2.9;  *p* = .12 | |
| **Iba1 Cell and Soma Analysis Summary:** | |  |  |  |  |  | |
| **Overall area occupied by cell (μm ^2^)** | **586.57**  **(15.01) ***** | **421.78**  **(16.73) ***** | **F [1,18] = 53.7;**  ***p* < .001** | 513.54  (18.8) | 507.05  (18.8) | F [1,40] = 0.1;  *p* = .81 | |
| Soma size perimeter (μm) | 30.89  (0.61) | 29.85  (0.62) | F [1,58] = 1.1;  *p* = .35 | 31.69  (0.92) | 30.39  (0.41) | F [1,72] = 1.6;  *p* = .21 | |
| Soma size area (μm ^2^) | 54.41  (1.67) | 51.27  (1.68) | F [1,51] = 1.8;  *p* = .19 | 59.57  (2.69) | 57.26  (1.2) | F [1,70] = 1.5;  *p* = .23 | |
| **n = cell/animal:** | 18/3 | 18/3 |  | 18/3 | 18/3 |  | |
| Data shown represents mean (± SEM); ** p* < .05; ** *p* < .01; *** *p* < .001 compared with control (adjusted *p* values)  Analyses: Repeated measures generalized linear mixed modelling with Bonferroni *post hoc* correction | | | | | | | |

**S. Figure 8. Effect of social isolation on GFAP positive cell characterisation in the dentate gyrus of the hippocampus.** Mean (± SEM) GFAP/SOX2 cell density stratified by cell type (Effect of exposure: F [1,56] = 4.5, *p* = .04; Effect of cell type: F [1,56] = 213.7, *p* < .001; Interaction: F [1,56] = 3.4, *p* = .08). Irrespective of exposure, all mice had significantly more radial glial cells, i.e., GFAP+/SOX2+ cells, in the dentate gyrus compared with GFAP positive astrocytes, i.e., GFAP+/SOX2- cells *** *p* < .001 (adjusted *p* values). Analyses: Two-way ANOVA with Bonferroni *post hoc* comparison.

**C**

**B**

**A**

**S. Figure 9. Effect of repeated injection, social isolation, and combined stress on hippocampal volume and proliferation (A)** mean (± SEM) hippocampal volume stratified by dorsal-ventral axis (Effect of exposure: F [3,92] = 1.0; *p* = .40; Effect of region: F [2,92] = 370.4; *p* < .001; Interaction: F [6,92] = 0.1; *p* > .99). For all groups, irrespective of exposure, the dorsal hippocampus was significantly smaller than the ventral hippocampus, a consequence of the way in which the tissue was sectioned, **(B)** mean (± SEM) Ki67 cell density in the dentate gyrus of the hippocampus (F [3,46] = 0.01, *p* > .99). There was no significant difference in Ki67 positive cell density across groups, **(C)** Representative photomicrograph taken from a control animal stained for Ki67 (40x magnification; scale bar = 40μm). *** *p* < .001 (adjusted *p* values). Analyses: One-way ANOVA with Bonferroni *post hoc* comparison or two-way ANOVA with Bonferroni *post hoc* comparison.

| **S. Table 6**. Effect of social hierarchy on altered neuroinflammatory outcomes | | | | | | |
| --- | --- | --- | --- | --- | --- | --- |
| **Variable** | **Control animals (n=20)** | | | **Physically stressed animals (n=10)** | | |
|  | Dominant Mean (SE) | Subordinate  Mean (SE) | *p* value | Dominant Mean (SE) | Subordinate  Mean (SE) | *p* value |
| **Iba1** |  |  |  |  |  |  |
| Immunoreactivity in the DG | 10.9 (0.3) | 12.7 (1.1) | 0.19 | 0.031 (0.004) | 0.032 (0.005) | 0.96 |
| Cell density in the dorsal DG | 15,860 (435.1) | 16,624 (2,203.1) | 0.76 | N/A | N/A | N/A |
| Cell density in the ventral DG | 11,581 (600.4) | 11,424  (1168.6) | 0.91 | 14,383  (706.7) | 12,615 (1413.1) | 0.34 |
| **GFAP** |  |  |  |  |  |  |
| Immunoreactivity in the DG | 34.7 (2.1) | 33.4 (1.6) | 0.64 | **29.3 (0.5)** | **33.5 (0.7)** | **0.008*** |
| Radial glial cell density in the DG | 35,410 (3,192.1) | 38,018 (6,927.6) | 0.75 | N/A | N/A | N/A |
| S100β cell density in the DG | 30,333 (6,156) | 25,523  (3,308) | 0.55 | N/A | N/A | N/A |
| * This finding does not alter the interpretation of the findings presented as GFAP was only further characterised in psychosocially stressed mice  Analyses: Independent samples *t* test or Mann Whitney test | | | | | | |

| **S. Table 7**. Effect of social hierarchy on altered neurogenesis outcomes | | | | | | |
| --- | --- | --- | --- | --- | --- | --- |
| **Variable** | **Control animals (n=20)** | | | **Physically stressed animals (n=10)** | | |
|  | Dominant Mean (SE) | Subordinate  Mean (SE) | *p* value | Dominant Mean (SE) | Subordinate  Mean (SE) | *p* value |
| DCX cell density in the DG | 39,839.5 (4,347.3) | 39,377.5 (5,508.4) | 0.95 | 24,280.7 (747.9) | 15,668.7 (1,924.9) | 0.014* |
| DCX cell density: proliferative stage neuroblasts | 12,271.8 (2258.5) | 16,893.8  (3,980) | 0.32 | 7,727.7 (702.5) | 4,782.8  (148.2) | 0.015* |
| DCX cell density: early post mitotic stage neuroblasts | 9,802.9 (2,263) | 7,137.1  (666.3) | 0.31 | 5,412.9 (556.5) | 2,078.2  (554.3) | 0.013* |
| DCX cell density: late post mitotic stage neuroblasts | 3,517.9 (716.8) | 3,496  (941.3) | 0.99 | 1,724.1 (765.4) | 1,251.9  (166) | 0.58 |
| Note: * Does not survive correction for multiple testing  Analyses: Independent samples *t* test or Mann Whitney test | | | | | | |

| **S.Table 8.** Summary of all neurobiological changes associated with chronic stress exposure | | | |
| --- | --- | --- | --- |
| **Variable** | **Physical stress** | **Psychosocial stress** | **Combined stress** |
| **Neuroinflammation readouts** |  |  |  |
| Iba1 positive cell immunoreactivity in the PFC | = | = | = |
| Iba1 positive cell immunoreactivity in the DG | **-** | **-** | = |
| Iba1 positive cell density in the DG | + | **-** | N/A |
| Iba1 positive cell morphology in the DG | b | c | N/A |
| GFAP positive cell immunoreactivity in the PFC | = | = | = |
| GFAP positive cell immunoreactivity in the DG | = | + | = |
| GFAP positive cell density in the DG | N/A | + | N/A |
| S100β positive cell density in the DG | N/A | + | N/A |
| **Neurogenesis measures** |  |  |  |
| Hippocampal volume | = | = | = |
| Overall Ki67 positive cell density in the DG | = | = | = |
| Overall: DCX positive cell density in the DG | **-** | = | **-** |
| Proliferative stage: DCX positive cell density in the DG | **-** | = | = |
| Intermediate stage: DCX positive cell density in the DG | = | = | = |
| Early post mitotic stage: DCX positive cell density in the DG | **-** | = | **-** |
| Late post mitotic stage: DCX positive cell density in the DG | = | + | = |
| Key:  + (shaded green); Represents a significant increase in readout relative to control  - (shaded blue); Represents a significant decrease in readout relative to control  = (unshaded); Represents no significant change in readout relative to control  a Represents a dystrophic morphology  b Represents a hyper ramified morphology | | | |

**REFERENCES**

1.	Deacon RMJ. Housing, husbandry and handling of rodents for behavioral experiments. Nat Protoc. 2006;1:936–946.

2.	Shimizu S. CHAPTER 32 – Routes of Administration. Lab. Mouse, 2004. p. 527–542.

3.	Van Loo PLP, et al. Long-term effects of husbandry procedures on stress-related parameters in male mice of two strains. Lab Anim. 2004;38:169–177.

4.	Bailey KR, Rustay NR, Crawley JN. Behavioral Phenotyping of Transgenic and Knockout Mice: Practical Concerns and Potential Pitfalls. ILAR J. 2006;47:124–131.

5.	Lad HV, et al. Behavioural battery testing: Evaluation and behavioural outcomes in 8 inbred mouse strains. Physiol Behav. 2010;99:301–316.

6.	Paylor R, Spencer CM, Yuva-Paylor LA, Pieke-Dahl S. The use of behavioral test batteries, II: Effect of test interval. Physiol Behav. 2006;87:95–102.

7.	Christmas AJ, Maxwell DR. A comparison of the effects of some benzodiazepines and other drugs on aggressive and exploratory behaviour in mice and rats. Neuropharmacology. 1970;9:17–29.

8.	Gould TD, Dao DT, Kovacsics CE. The open field test. Mood anxiety Relat. phenotypes mice Charact. using Behav. tests., Gould, Todd D.: Department of Psychiatry, Mood and Anxiety Disorders Program, University of Maryland School of Medicine, 685 W. Baltimore Street, MSTF, Rm934D, Baltimore, MD, US, 21201, Tgould@psych.Umaryland.Edu: Humana Press; 2009. p. 1–20.

9.	Powell TR, Fernandes C, Schalkwyk LC. Depression-Related Behavioral Tests. Curr Protoc Mouse Biol. 2012. 2012. https://doi.org/10.1002/9780470942390.mo110176.

10.	Samuels BA, Hen R. Novelty-Suppressed Feeding in the Mouse. In: Gould TD, editor. Mood Anxiety Relat. Phenotypes Mice Charact. Using Behav. Tests, Vol. II, Totowa, NJ: Humana Press; 2011. p. 107–121.

11.	Papp M, Willner P, Muscat R. An animal model of anhedonia: attenuation of sucrose consumption and place preference conditioning by chronic unpredictable mild stress. Psychopharmacology (Berl). 1991;104:255–259.

12.	Willner P, Towell A, Sampson D, Sophokleous S, Muscat R. Reduction of sucrose preference by chronic unpredictable mild stress, and its restoration by a tricyclic antidepressant. Psychopharmacology (Berl). 1987;93:358–364.

13.	Pyndt Jørgensen B, et al. A possible link between food and mood: dietary impact on gut microbiota and behavior in BALB/c mice. PLoS One. 2014;9:e103398.

14.	Strekalova T, Steinbusch HWM. Measuring behavior in mice with chronic stress depression paradigm. Prog Neuro-Psychopharmacology Biol Psychiatry. 2010;34:348–361.

15.	Lucki I, Dalvi A, Mayorga AJ. Sensitivity to the effects of pharmacologically selective antidepressants in different strains of mice. Psychopharmacology (Berl). 2001;155:315–322.

16.	Porsolt RD, Anton G, Blavet N, Jalfre M. Behavioural despair in rats: a new model sensitive to antidepressant treatments. Eur J Pharmacol. 1978;47:379–391.

17.	Lindzey G, Manosevitz M, Winston H. Social dominance in the mouse. Psychon Sci. 1966;5:451–452.

18.	Yang M, Crawley JN. Simple behavioral assessment of mouse olfaction. Curr Protoc Neurosci. 2009;Chapter 8:Unit-8.24.

19.	Davidson JM, Jones LE, Levine S. Feedback Regulation of Adrenocorticotropin Secretion in “Basal” and “Stress” Conditions: Acute and Chronic Effects of Intrahypothalamic Corticoid Implantation. Endocrinology. 1968;82:655–663.

20.	Sadler AM, Bailey SJ. Validation of a refined technique for taking repeated blood samples from juvenile and adult mice. Lab Anim. 2013;47:316–319.

21.	Bielohuby M, Popp S, Bidlingmaier M. A guide for measurement of circulating metabolic hormones in rodents: Pitfalls during the pre-analytical phase. Mol Metab. 2012;1:47–60.

22.	Gage GJ, Kipke DR, Shain W. Whole animal perfusion fixation for rodents. J Vis Exp. 2012:3564.

23.	Kielar C, et al. Successive neuron loss in the thalamus and cortex in a mouse model of infantile neuronal ceroid lipofuscinosis. Neurobiol Dis. 2007;25:150–162.

24.	Bible E, Gupta P, Hofmann SL, Cooper JD. Regional and cellular neuropathology in the palmitoyl protein thioesterase-1 null mutant mouse model of infantile neuronal ceroid lipofuscinosis. Neurobiol Dis. 2004;16:346–359.

25.	Pereira Dias G, et al. Consequences of cancer treatments on adult hippocampal neurogenesis: implications for cognitive function and depressive symptoms. Neuro Oncol. 2014;16:476–492.

26.	Gundersen HJG. Notes on the estimation of the numerical density of arbitrary profiles: the edge effect. J Microsc. 1977;111:219–223.

27.	Gundersen HJG, et al. Some new, simple and efficient stereological methods and their use in pathological research and diagnosis. APMIS. 1988;96:379–394.

28.	Slomianka L, West MJ. Estimators of the precision of stereological estimates: An example based on the CA1 pyramidal cell layer of rats. Neuroscience. 2005;136:757–767.

29.	Gundersen HJG, Jensen EB V, Kieu K, Nielsen J. The efficiency of systematic sampling in stereology — reconsidered. J Microsc. 1999;193:199–211.

30.	Schneider CA, Rasband WS, Eliceiri KW. NIH Image to ImageJ: 25 years of image analysis. Nat Methods. 2012;9:671–675.

31.	Cătălin B, Alexandru D, Albu C, Iancău M. Microglia branching using a Sholl analysis method. Curr Heal Sci J. 2015:1–5.

32.	Plümpe T, et al. Variability of doublecortin-associated dendrite maturation in adult hippocampal neurogenesis is independent of the regulation of precursor cell proliferation. BMC Neurosci. 2006;7:77.

33.	Priebe K, et al. Maternal influences on adult stress and anxiety-like behavior in C57BL/6J and BALB/cJ mice: A cross-fostering study. Dev Psychobiol. 2005;47:398–407.

34.	Tang X, Orchard SM, Sanford LD. Home cage activity and behavioral performance in inbred and hybrid mice. Behav Brain Res. 2002;136:555–569.

35.	Crawley JN, et al. Behavioral phenotypes of inbred mouse strains: implications and recommendations for molecular studies. Psychopharmacology (Berl). 1997;132:107–124.

36.	Kessler RC, et al. Lifetime Prevalence and Age-of-Onset Distributions of DSM-IV Disorders in the National Comorbidity Survey Replication. Arch Gen Psychiatry. 2005;62:593–602.

37.	Bogren M, Brådvik L, Holmstrand C, Nöbbelin L, Mattisson C. Gender differences in subtypes of depression by first incidence and age of onset: a follow-up of the Lundby population. Eur Arch Psychiatry Clin Neurosci. 2018;268:179–189.

38.	Klein DN, et al. Predictors of first lifetime onset of major depressive disorder in young adulthood. J Abnorm Psychol. 2013;122:1–6.
